# A Chemically Characterized Polyherbal Extract Selectively Attenuates Chronic Unpredictable Stress-Induced Redox-Sensitive, Inflammatory, and Metabolic Alterations in Female Rats

**DOI:** 10.3390/ijms27188298

**Published:** 2026-09-17

**Authors:** Sanja Kovačević, Ljupka Gligorovska, Biljana Bursać, Mirna Jovanović, Danijela Vojnović Milutinović, Srdjan Kesić, Branka Petković, Uroš Gašić, Ana Ćirić, Dejan Stojković, Ana Djordjevic

**Affiliations:** 1 Department of Biochemistry, Institute for Biological Research “Siniša Stanković”, National Institute of the Republic of Serbia, University of Belgrade, 142 Despot Stefan Blvd, 11108 Belgrade, Serbia; sanja.kovacevic@ibiss.bg.ac.rs (S.K.); ljupkag@gmail.com (L.G.); bbursac@ibiss.bg.ac.rs (B.B.); mirna.jovanovic@gmail.com (M.J.); dvojnovic@ibiss.bg.ac.rs (D.V.M.); 2 Department of Neurophysiology, Institute for Biological Research “Siniša Stanković”, National Institute of the Republic of Serbia, University of Belgrade, 142 Despot Stefan Blvd, 11108 Belgrade, Serbia; srdjan.kesic@ibiss.bg.ac.rs (S.K.); janac@ibiss.bg.ac.rs (B.P.); 3 Department of Plant Physiology, Institute for Biological Research “Siniša Stanković”, National Institute of the Republic of Serbia, University of Belgrade, 142 Despot Stefan Blvd, 11108 Belgrade, Serbia; uros.gasic@ibiss.bg.ac.rs (U.G.); rancic@ibiss.bg.ac.rs (A.Ć.); dejanbio@ibiss.bg.ac.rs (D.S.)

**Keywords:** *Centaurium erythraea*, *Valeriana officinalis*, *Melissa officinalis*, *Humulus lupulus*, chronic stress, female rats, oxidative stress, inflammation

## Abstract

Chronic stress contributes to metabolic and mental disorders, with accumulating evidence suggesting differences in stress responses between females and males. Although *Centaurium erythraea*, *Valeriana officinalis*, *Melissa officinalis*, and *Humulus lupulus* possess well-documented antioxidant, anti-inflammatory, and neuroactive properties, the biological activity of their combined extract remains largely unexplored. Here, a chemically characterized polyherbal extract was evaluated for in vitro biosafety and its effects on chronic unpredictable stress-induced behavioral, metabolic, and molecular alterations in male and female rats. LC-HRMS/MS identified 121 metabolites, including phenolic acids, iridoid glycosides, flavonoids, and xanthone derivatives, while the extract showed a favorable biosafety profile in HaCaT keratinocytes. Chronic stress-induced alterations were predominantly observed in females, including reduced body mass, serum glucose and insulin levels, enhanced hippocampal glucocorticoid signaling, increased NRF2 and NFκB protein levels, altered IRS1 signaling, and anxiety-like behavior. Treatment with the extract normalized glucose tolerance, restored hippocampal IRS1, phosphorylated IRS1 (Ser307), NRF2, and NFκB protein levels in females. These findings indicate that, under the experimental conditions employed, chronic stress-associated alterations and the effects of the polyherbal extract were predominantly observed in female rats, and suggest that the extract may mitigate stress-induced metabolic disturbances through modulation of redox-sensitive, inflammatory, and hippocampal insulin signaling pathways.

## 1. Introduction

Chronic stress is an increasingly prevalent issue in modern society, where individuals are regularly exposed to various psychological and environmental stressors. Prolonged exposure to such stressors has been shown to contribute to the onset of numerous physical and mental health disorders, including anxiety, depression and metabolic disorders [1]. Clinical and animal studies suggest that responses to chronic stress may differ between females and males, potentially due to differences in hormonal regulation and stress-response mechanisms [2,3]. One of the principal neuroendocrine systems activated by chronic stress is the hypothalamic–pituitary–adrenal (HPA) axis, which stimulates glucocorticoid secretion and regulates stress responses in both peripheral tissues and the brain [4]. In peripheral tissues, inactive glucocorticoids are converted to their active forms (cortisol in humans and corticosterone in rodents) through prereceptor metabolism mediated by the enzymes 11β hydroxysteroid dehydrogenase type 1 (11βHSD1) and hexose-6-phosphate dehydrogenase (H6PDH) [5]. Glucocorticoids exert their effects by binding to the glucocorticoid receptor (GR), a hormone-dependent transcriptional regulator that is abundantly expressed in the hippocampus, a part of the limbic system important for memory and learning [6]. The transcriptional activity of GR is further regulated by site-specific phosphorylation, with phosphorylation at Ser 211 associated with enhanced GR transcriptional activity and nuclear signaling, whereas phosphorylation at Ser 226 is linked to reduced transcriptional efficacy and receptor nuclear export. Dysregulation of the GR or hypercortisolemia can lead to altered hippocampal function, manifested by increased anxiety-like behaviors, but also to stress-induced metabolic dysfunction, which contributes to the development of systemic and hippocampal insulin resistance. Chronic stress or chronic administration of glucocorticoids disrupts the HPA system and leads to repeated or sustained glucocorticoid exposure, hyperglycemia and hyperinsulinemia, which subsequently leads to hippocampal degeneration, memory and learning deficits, and depressive- and anxiety-like behaviors [6,7]. The mechanisms underlying the relationship between chronic stress, insulin resistance and stress-related disorders are still unclear, and understanding the molecular signaling pathways that are activated in response to stress is very important for the development of pharmacological interventions to prevent and treat stress-related diseases. One of the potential mechanisms of stress-related disorders is oxidative damage in the hippocampus resulting from a redox imbalance in the production of reactive oxygen species (ROS) and the activity of the antioxidant system [8,9]. Nuclear factor erythroid 2-related factor 2 (NRF2) is a transcription factor that regulates a number of genes that protect cells against the damaging effects of oxidative and environmental stress [10]. NRF2 is expressed in all cell types, and under unstressed conditions, the amount of its basal protein is usually low as it is sequestered by Kelch-like ECH-associated protein 1 (KEAP1) and subsequently ubiquitinated and proteasomally degraded [11,12]. Under stress conditions, the KEAP1-NRF2 complex is disrupted and NRF2 is activated. Activation of NRF2 leads to its accumulation in the nucleus, where it acts as a transcription factor for genes encoding antioxidant enzymes such as superoxide dismutase 1 (SOD1), glutathione peroxidase (GPx), glutathione reductase (GRS) and catalase (CAT) [11]. These cytoprotective enzymes, encoded by NRF2 target genes, are essential for protection against a variety of oxidative damage and thus for many diseases in which oxidative stress is a central pathological feature, including metabolic syndrome, type 2 diabetes, neuronal degeneration and even cancer.

As the global burden of stress-related diseases continues to increase and chemical medications are often ineffective and have considerable side effects, there is a growing need for safer and more effective therapeutic strategies to alleviate the negative effects of chronic stress and improve overall physical and mental well-being. Medicinal plants such as *Valeriana officinalis* (valerian), *Melissa officinalis* (lemon balm), *Humulus lupulus* (common hop) and *Centaurium erythraea* (common centaury) have long been used in traditional medicine for their therapeutic properties, particularly their calming and soothing effects. These herbs are known for their anxiolytic, sedative and antioxidant properties, which can help counteract the negative effects of chronic stress by regulating behavioral responses, improving hormonal balance and enhancing antioxidant defense systems [13,14]. *Valeriana officinalis* is widely used in traditional medicine for its anxiolytic and sedative effects, but there are numerous studies showing that valerian extract has antioxidant and neuroprotective properties against neuronal degeneration and oxidative stress in the hippocampus [15,16]. Like valerian, *Melissa officinalis* has been extensively studied for its anxiolytic and antidepressant properties, as its extract was found to improve anxiety- and depression-like behavior in a mouse model of restraint stress-induced depression [17,18]. These effects were attributed to the reduction in oxidative stress, restoration of serum corticosterone levels and modulation of apoptosis markers in the hippocampus and prefrontal cortex, indicating the neuroprotective effect of *Melissa officinalis* extract [18]. Hop (*Humulus lupulus*) is traditionally used for its medicinal properties, particularly for relieving symptoms of anxiety, stress and depression, but it is also known for its anti-inflammatory, antioxidant and antimicrobial effects [19]. Recent in vivo/vitro and clinical studies have demonstrated the positive effects of hop bioactive compounds on metabolic disorders by influencing body weight, lipid metabolism, glucose homeostasis and insulin resistance [19,20]. *Centaurium erythraea* is traditionally used as diabetes treatment for its anti-diabetic, anti-inflammatory and antioxidant properties. Animal studies have shown that *Centaurium erythraea* extract helps to normalize glucose levels by stimulating its elimination in the intestine and increasing insulin levels in the blood [21,22]. In addition, treatment with *Centaurium erythraea* extract has shown protective properties against oxidative stress by scavenging superoxide anions and hydroxyl radicals and stimulating the activity of antioxidant enzymes in the kidneys, liver and serum [22,23].

To explore the potential of these medicinal plants in alleviating stress-related symptoms, we prepared a combined extract of *Centaurium erythraea*, *Valeriana officinalis*, *Melissa officinalis*, and *Humulus lupulus*. Given the evidence that responses to chronic stress may differ between females and males, we investigated the effects of this polyherbal extract (hereafter referred to as the extract) in both female and male Wistar rats using an established and well-characterized model of chronic unpredictable stress [4]. We evaluated behavioral responses using the open field test (OFT) and elevated plus maze (EPM) and assessed systemic and hippocampal insulin signaling, glucocorticoid signaling, inflammation, and antioxidant defense.

## 2. Results

### 2.1. Chemical Profile of Polyherbal Mixture Extract

UHPLC-Orbitrap HRMS profiling demonstrated that the polyherbal extract was chemically diverse, with 121 metabolites tentatively identified based on their retention times, accurate masses, molecular formulae, mass errors, and MS/MS fragmentation patterns. The low mass deviations, generally within approximately ±2 ppm, support a high level of confidence in the assigned elemental compositions. Identified compounds were classified into the following nine major chemical groups: hydroxybenzoic acid derivatives, hydroxycinnamic acid derivatives, iridoid glycosides, flavan-3-ol gallates, flavonoid *O*-glycosides, flavonoid *C*-glycosides, flavonoid aglycones, xanthone glycosides, and xanthone aglycones (Supplementary Table S1). A detailed description of the phytochemical composition and representative fragmentation characteristics is provided in Supplementary Note S1.

The semi-quantitative analysis revealed marked differences in the estimated contents of individual metabolites within the respective phytochemical classes (Table 1). Among the selected compounds, hydroxycinnamic acid derivatives showed particularly high estimated contents, with sagerinic acid (17,774.56 mg/kg) and rosmarinic acid (10,471.47 mg/kg) showing the highest estimated contents within this class, followed by lithospermic acid (7511.84 mg/kg) and schizotenuin A (6904.05 mg/kg). Iridoid glycosides were also strongly represented, particularly caffeoyl-loganin (8113.97 mg/kg), alpigenoside (5811.64 mg/kg), and 1-*O*-hexosyl-loganic acid (Asystasioside A; 2188.86 mg/kg). Hydroxybenzoyl-galloyl hexoside was a prominent hydroxybenzoic acid derivative (5058.69 mg/kg). Selected flavonoid and xanthone derivatives showed lower semi-quantitative estimates. Among the selected flavonoids, hispidulin and apigenin 8-C-hexoside showed contents of 291.21 and 277.91 mg/kg, respectively, while representative xanthone derivatives included trihydroxy-dimethoxy xanthone O-rhamnosyl-hexoside (166.41 mg/kg) and swertianin (83.10 mg/kg).

### 2.2. In Vitro Biosafety

**Figure 1:**
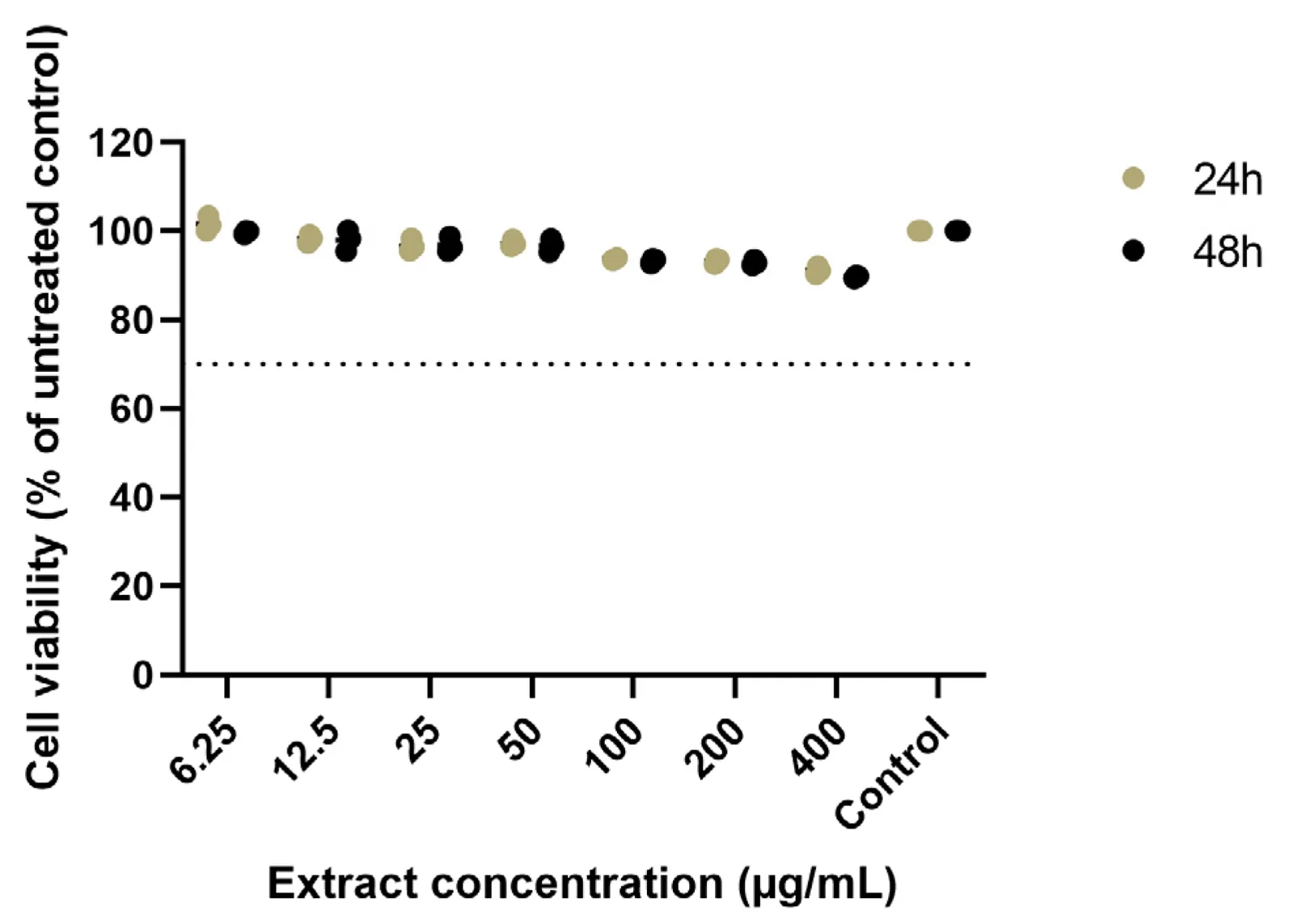
Effect of the polyherbal extract on the viability of HaCaT human keratinocytes after 24 and 48 h of exposure. Cell viability was determined using the crystal violet assay and is expressed as a percentage relative to the untreated control (100%). The dashed horizontal line represents the 70% viability threshold defined by ISO 10993-5 for the classification of non-cytotoxic materials. Data are presented as mean ± SD of three independent experiments (*n* = 3), each performed in triplicate wells. Technical replicates were averaged within each independent experiment before statistical analysis.

The biosafety of the polyherbal extract was evaluated in HaCaT human keratinocytes after 24 and 48 h of treatment at concentrations ranging from 6.25 to 400 µg/mL. Cell viability remained high throughout the tested concentration range, ranging from 91.13% to 101.52% after 24 h and from 89.73% to 99.73% after 48 h (Figure 1). At 24 h, treatment with 6.25 and 12.5 µg/mL did not significantly affect cell viability compared with the untreated control (adjusted *p* = 0.3943 and 0.2500, respectively). A statistically significant decrease was first observed at 25 µg/mL (adjusted *p* = 0.0051), followed by 50 µg/mL (adjusted *p* = 0.0217), while all higher concentrations (100–400 µg/mL) also differed significantly from the control (adjusted *p* < 0.0001). A similar trend was observed after 48 h of exposure. Cell viability at 6.25 and 12.5 µg/mL remained comparable to the control (adjusted *p* = 0.9996 and 0.1306, respectively), whereas significant reductions were detected at 25 µg/mL (adjusted *p* = 0.0072), 50 µg/mL (adjusted *p* = 0.0055), and at concentrations of 100–400 µg/mL (all adjusted *p* < 0.0001). Although these differences reached statistical significance, the decrease in viability was modest, with the lowest value remaining at 89.73% after 48 h of treatment. Importantly, cell viability exceeded the 70% threshold recommended by ISO 10993-5 at all tested concentrations and exposure times, indicating that the extract did not exhibit cytotoxic effects toward HaCaT keratinocytes under the experimental conditions used.

### 2.3. Food and Energy Intake and Body Mass

The effects of stress can be evaluated by monitoring the body mass of the stressed animals. Although all animal groups had unchanged food and caloric intake compared to the control group, the female rats showed reduced body mass regardless of the extract treatment (Table 2, S vs. C, ** *p* < 0.01, S + E vs. C, and * *p* < 0.05). On the other hand, stress-exposed male rats treated with extract showed decreased energy intake in comparison to stressed rats (Table 2, ^#^ *p* < 0.05, and S + E vs. S), while their energy intake was decreased in comparison to unstressed controls (Table 2, * *p* < 0.05, and S + E vs. C). Food intake and body mass remained unchanged.

### 2.4. Behavioral Tests

#### 2.4.1. Open Field Test

**Figure 2:**
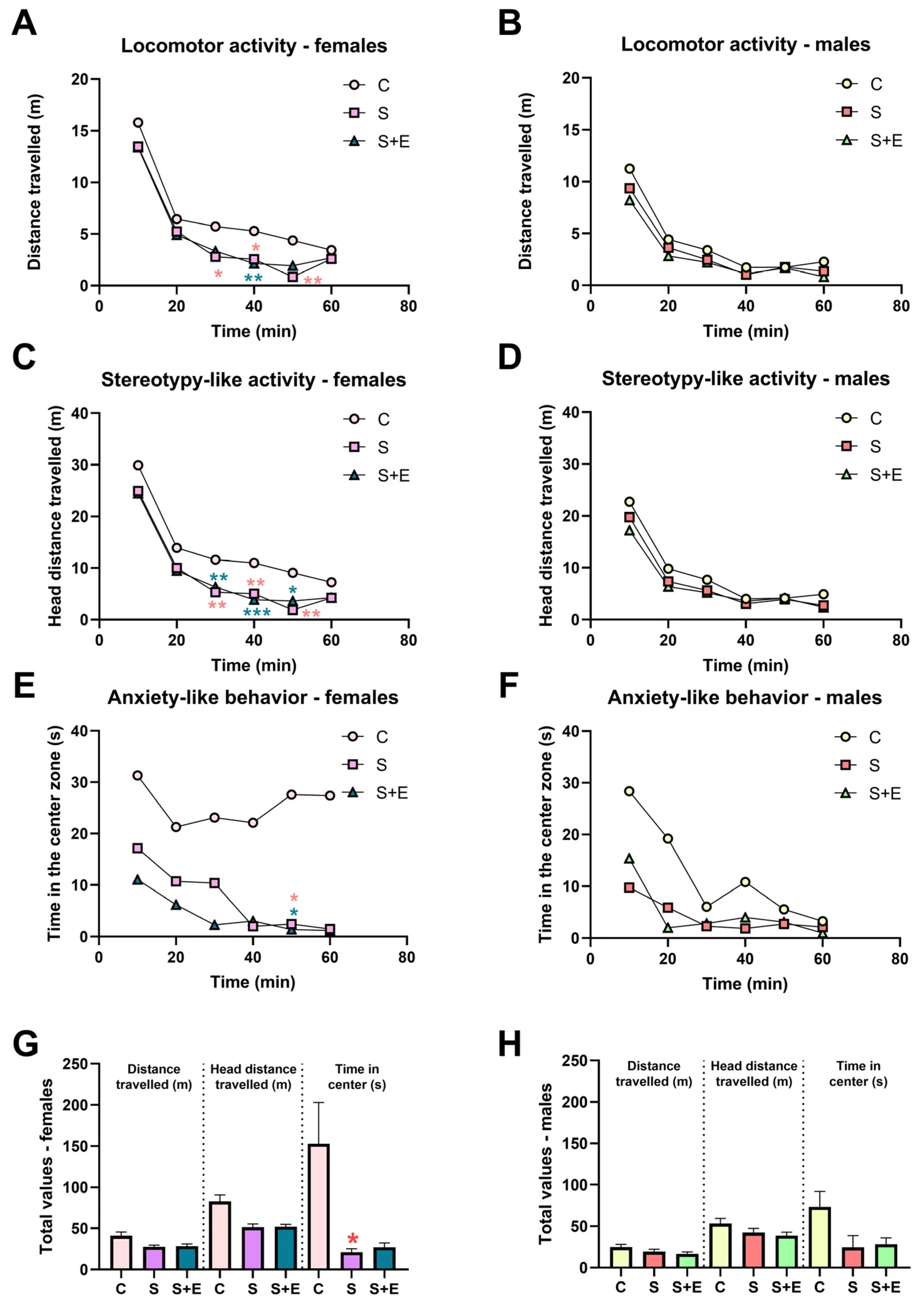
Behavioral response to chronic stress with and without an herbal extract mixture in female and male rats assessed in the open field test. Locomotor activity (**A**,**B**), stereotypy-like activity (**C**,**D**), and time spent in the central zone as a measure of anxiety-like behavior (**E**,**F**) were evaluated in female and male control (C), stressed (S), and stressed rats receiving the herbal extract mixture (S + E). Time-course data are presented as mean ± SEM, whereas total values for locomotor activity, stereotypy-like activity and time spent in the central zone are shown in panels (**G**,**H**) as mean ± SEM. Significant differences, between groups resulting from one-way ANOVA followed by Tukey’s post hoc test or non-parametric Kruskal–Wallis *H* test followed by Dunn’s post hoc test, are indicated as follows: * *p* < 0.05, ** *p* < 0.01, *** *p* < 0.001 (S and S + E vs. C).

In both sexes, locomotor activity tended to decrease over 60 min, representing a typical intra-session habituation response. In females exposed to stress, locomotor activity dropped significantly from 30 to 50 min compared to control females (* *p* < 0.05, for 30 and 40 min; ** *p* < 0.01 for 50 min), reaching control-like level during the last 10 min of the testing session (Figure 2). However, in the group of females exposed to stress and treated with the extract, a statistically significant difference compared to the control group was observed only in the fourth 10 min set, which corresponds to the 40 min mark on the graph (** *p* < 0.01). There was no significant difference between the group exposed to stress and to the combination of stress and extract (Figure 2). Despite a tendency to decrease, locomotor activity in stressed males, with or without extract treatment did not differ significantly from that of control males.

Stereotypy-like activity generally followed locomotor activity patterns and was typically higher in females than in males. In females, stereotypy-like activity was significantly reduced in stressed animals, regardless of extract treatment, compared to control females from 30 to 50 min (for 30 min-** *p* < 0.01, S vs. C and S + E vs. C; for 40 min-** *p* < 0.01, S vs. C, *** *p* < 0.001, and S + E vs. C; and for 50 min-** *p* < 0.01, S vs. C, * *p* < 0.05, and S + E vs. C) indicating stress-related differences but no effects attributable to the extract. In males, no significant differences were observed among the three groups tested (Figure 2).

The OFT revealed notable findings regarding stress-exposure associated thigmotactic behavior, i.e., avoidance of the central zone of the open-field arena. Stressed females, with or without extract treatment, spent less time in the central zone of the arena compared to the control females, at the 50 min time point (* *p* < 0.05, S vs. C and S + E vs. C) (Figure 2). Stressed males, with or without extract treatment, spent less time in the central zone than control males, but this difference was not significant, as well as slight differences between S + E and S groups of males. Analyzing total values of the above mentioned parameters no significant differences between the groups were obtained in rats of both sexes, except for the group of stressed females, which showed a decrease compared to corresponding control group regarding time spent in the central zone of the test arena (* *p* < 0.05) (Figure 2).

#### 2.4.2. Elevated Plus Maze

**Figure 3:**
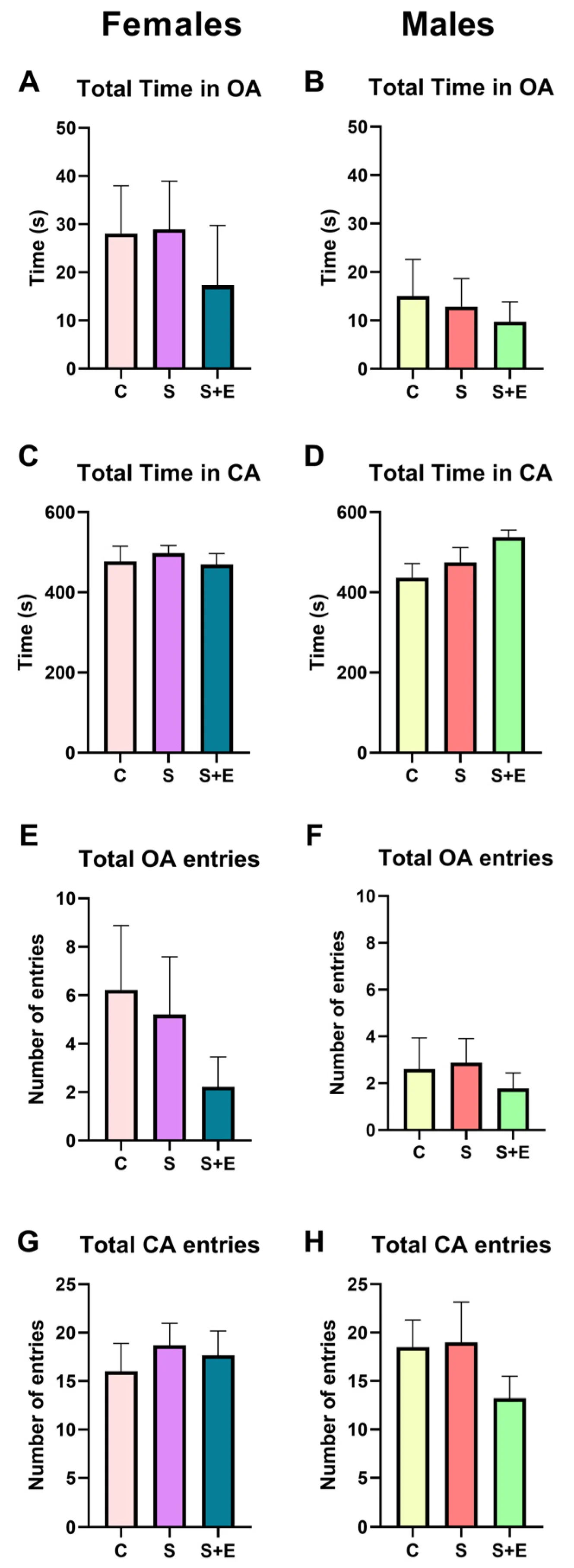
Behavioral response to chronic stress with and without herbal extract mixture in female and male rats assessed in the elevated plus maze test. Total time spent in the open arms (**A**,**B**) and closed arms (**C**,**D**), as well as the total number of entries into the open arms (**E**,**F**) and closed arms (**G**,**H**), were evaluated in female and male control (C), stressed (S), and stressed rats receiving the herbal extract mixture (S + E). Data are presented as mean ± SEM. OA—open arm, CA—closed arm.

Testing in the EPM did not reveal any significant changes associated with anxiety-like behavior. However, this does not mean there are no variations in EPM indices among the tested groups. In females, total time in the open arms (OAs) decreased in stressed animals with extract treatment, as did the number of OA entries, regardless of extract treatment, while total time in the closed arms (CAs) and the number of CA entries remained relatively constant across all three groups. In males, total time in the open arms (OAs) and the number of OA entries decreased, while total time in the closed arms (CAs) increased, along with a decrease in the number of CA entries, in stressed animals with extract treatment compared to control animals (Figure 3).

### 2.5. Measurements of Systemic Insulin Sensitivity Parameters

**Figure 4:**
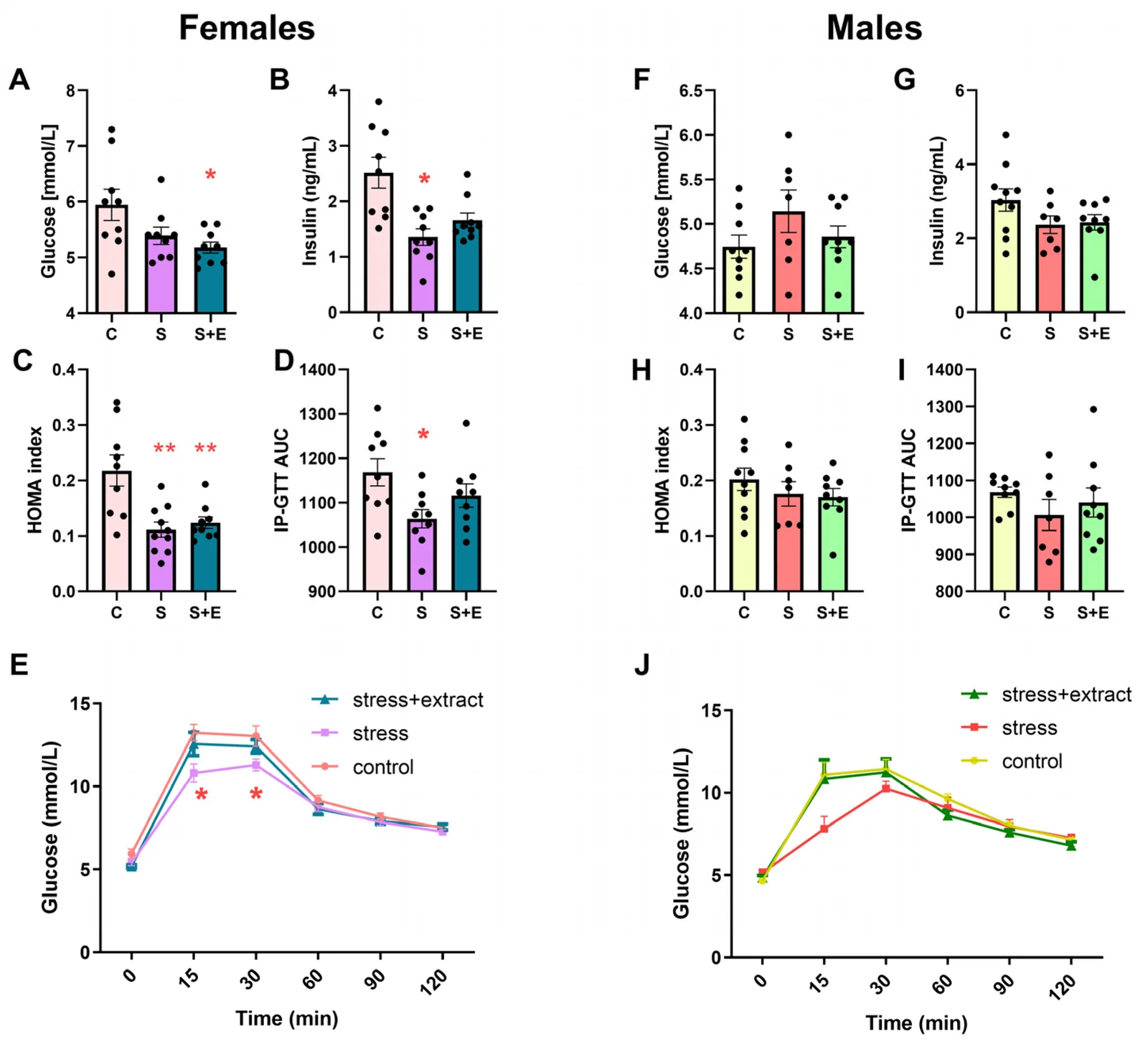
Systemic insulin sensitivity in female and male rats. The effects of the chronic stress and combination of stress and plant extracts on systemic insulin sensitivity were evaluated by measuring the glucose (**A**,**F**) and insulin (**B**,**G**) concentration in the blood, calculating HOMA index (**C**,**H**) and performing an intraperitoneal glucose tolerance test (IP-GTT) (**E**,**J**) whose result was AUC (**D**,**I**) in female and male rats. The values represent the mean ± SEM (*n* = 7–9 animals per group). Significant differences, between groups resulting from one-way ANOVA followed by Tukey’s post hoc test or non-parametric Kruskal–Wallis *H* test followed by Dunn’s post hoc test, are indicated as follows: * *p* < 0.05, S and S + E vs. C; ** *p* < 0.01, S and S + E vs. C.

The effects of the chronic stress and combination of plant extracts on systemic insulin sensitivity were evaluated by measuring the insulin and glucose concentration in the blood, calculating homeostasis model assessment (HOMA) index, and performing an intraperitoneal glucose tolerance test (IP-GTT) (Figure 4). The extract showed a statistically significant effect on lowering the blood glucose concentration in the stressed females receiving the extract in comparison to the control group (* *p* < 0.05). In addition, the stress itself lowered the insulin concentration in the blood compared to the female control animals (* *p* < 0.05), while treatment with the extract normalized the insulin level to the control level. Both treatments led to a statistically significant decrease in the HOMA index compared to the female control animals (** *p* < 0.01). Further we performed the IP-GTT, whose result—area under the curve (AUC)—represents an indirect measure of systemic insulin resistance. As can be seen in Figure 4, stress led to a statistically significant decrease in the AUC value in the stressed female rats compared to the control group of animals (* *p* < 0.05). As in the case of insulin, the AUC value in the female extract-treated group was normalized to the level of the control group. In addition, during IP-GTT, chronic stress significantly decreased glucose level in 15 and 30 min (* *p* < 0.05), while extract returned the glucose values closer to unstressed female rats. There were no statistically significant changes in either of the investigating parameters in male rats exposed to chronic stress with or without extract (Figure 4).

### 2.6. Insulin Signaling in the Hippocampus

**Figure 5:**
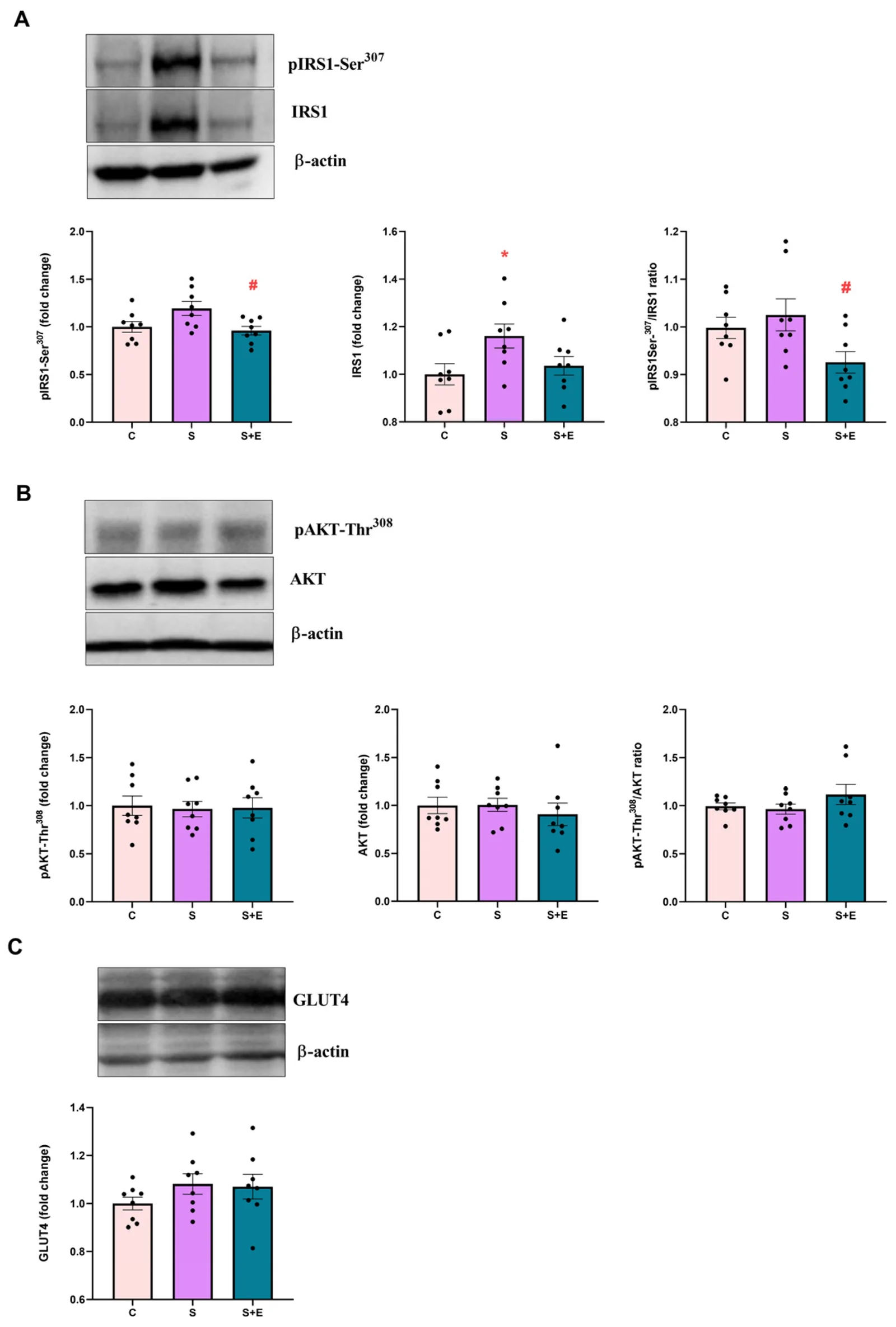
Insulin signaling pathway in the hippocampus of female rats. Representative Western blots and protein levels of total IRS1, pIRS1-Ser307 and their ratio (**A**) total AKT, pAKT-Thr308 and their ratio (**B**) and GLUT4 (**C**). The values represent the mean ± SEM (*n* = 7–9 animals per group) for each protein normalized to β-actin. Significant differences, between groups resulting from one-way ANOVA followed by Tukey’s post hoc test or non-parametric Kruskal–Wallis *H* test followed by Dunn’s post hoc test, are indicated as follows: * *p* < 0.05, S vs. C; ^#^ *p* < 0.05, S + E vs. S.

Effects of chronic stress and/or treatment with extract on hippocampal insulin signaling pathway were assessed using protein levels of IRS1, its negative phosphorylation form pIRS1-Ser^307^, AKT, its activating phosphorylation form pAKT-Thr^308^ and main glucose cell transporter GLUT4. Our results showed that chronic stress increased level of total IRS1 (* *p* < 0.05) in female rats while extract treatment returned its level closer to control value. Extract significantly decreased level of pIRS1-Ser^307^ (^#^ *p* < 0.05) as well as ratio of pIRS1-Ser^307^ (^#^ *p* < 0.05) to total IRS1 in comparison to stressed group of female rats. There were no changes in AKT, pAKT-Thr^308^ and GLUT4 regardless of the treatment (Figure 5).

**Figure 6:**
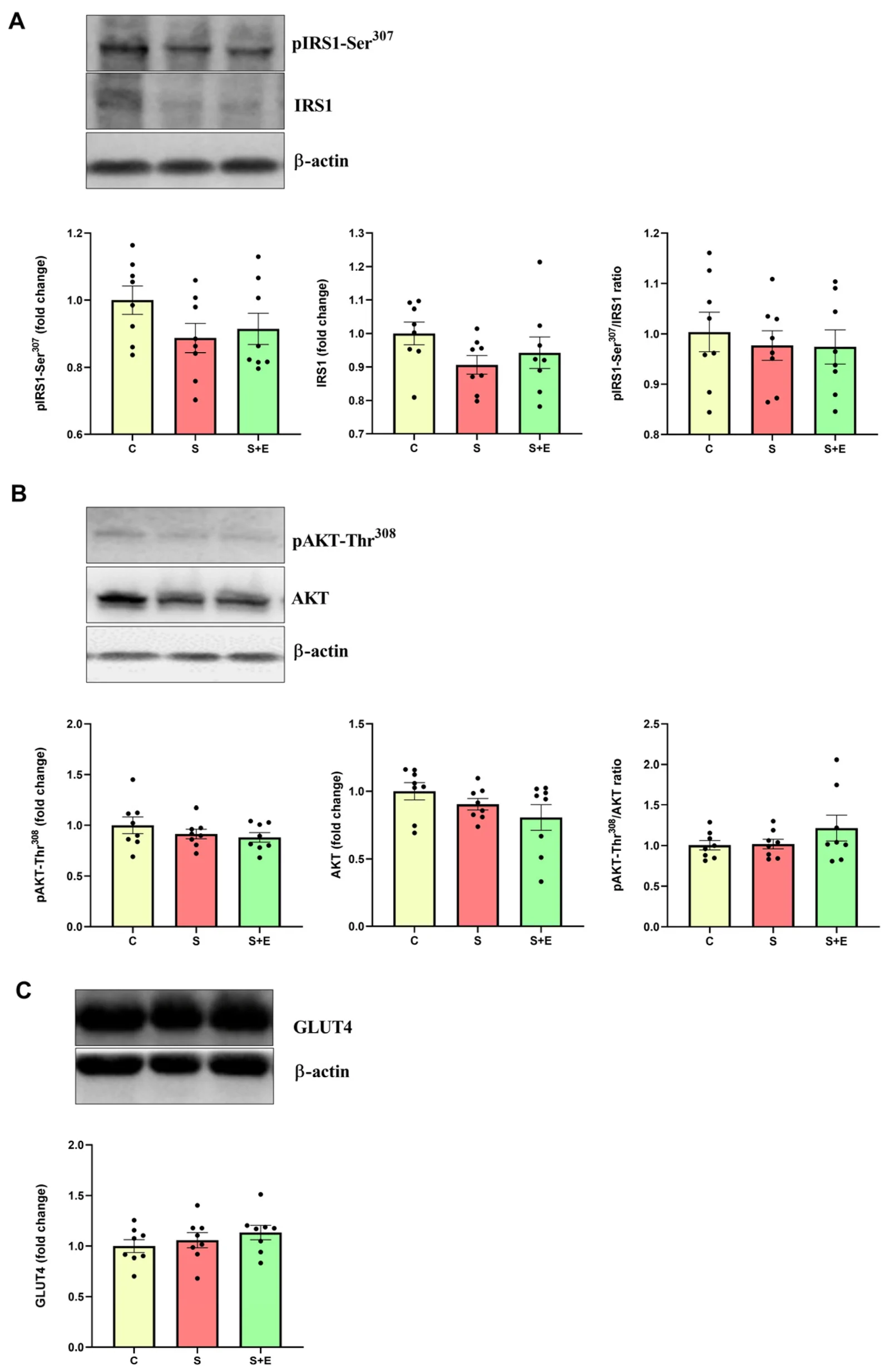
Insulin signaling pathway in the hippocampus of male rats. Representative Western blots and protein levels of total IRS1, pIRS1-Ser307 and their ratio (**A**) total AKT, pAKT-Thr308 and their ratio (**B**) and GLUT4 (**C**). The values represent the mean ± SEM (*n* = 7–9 animals per group) for each protein normalized to β-actin.

Analyzed insulin signaling parameters remained unchanged in male rats, regardless of the treatment (Figure 6).

### 2.7. Glucocorticoid Signaling Pathway

**Figure 7:**
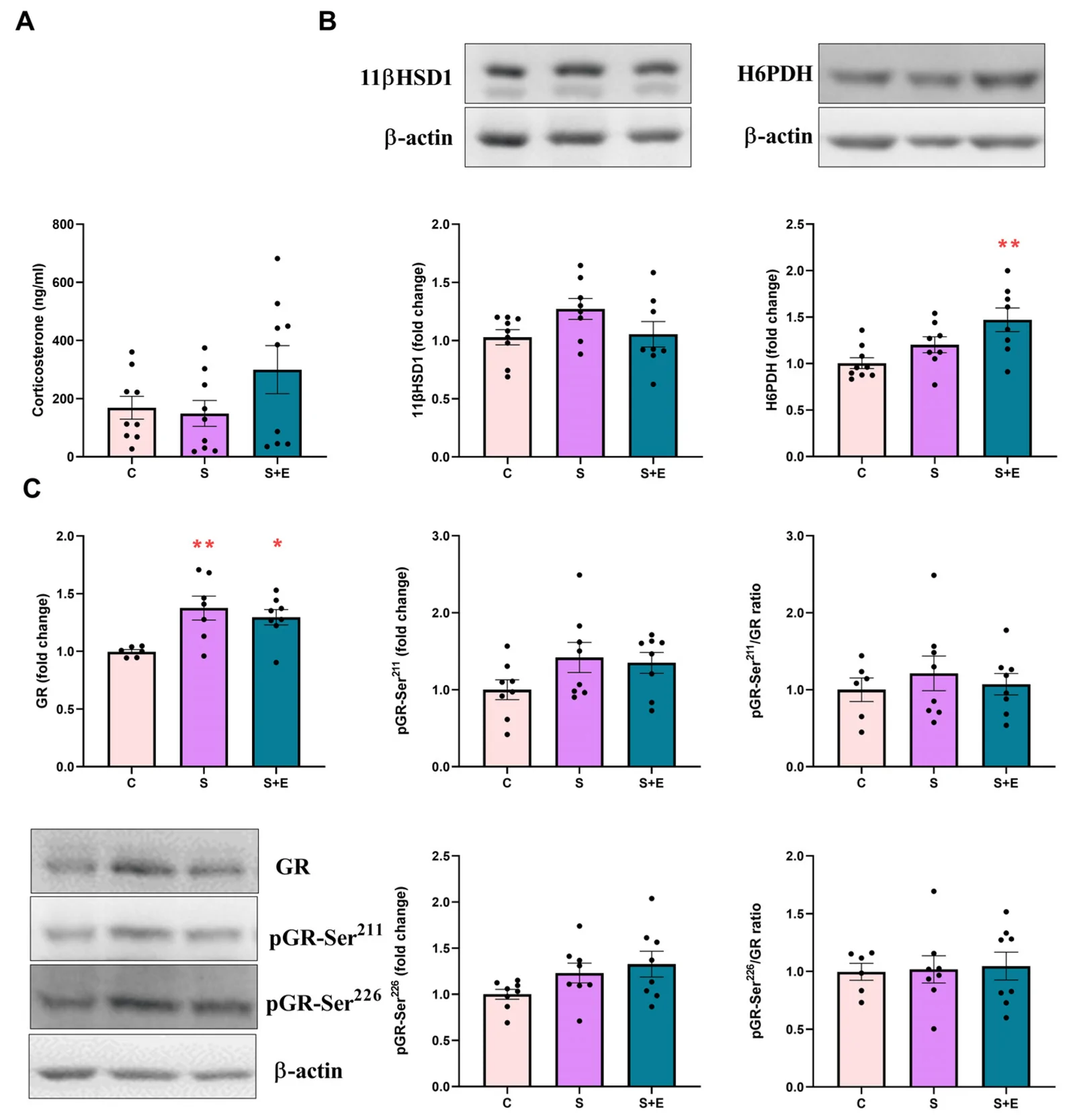
Glucocorticoid signaling pathway in the hippocampus of female rats. Corticosterone concentration (**A**), representative Western blots and protein levels of 11βHSD1 and H6PDH (**B**), GR, pGR-Ser^226^, pGR-Ser^211^ and ratio of phosphorylated forms to total GR (**C**). The values represent the mean ± SEM (*n* = 7–9 animals per group) for each protein normalized to β-actin. Significant differences, between groups resulting from one-way ANOVA followed by Tukey’s post hoc test or non-parametric Kruskal–Wallis *H* test followed by Dunn’s post hoc test, as appropriate, are indicated as follows: * *p* < 0.05; ** *p* < 0.01.

The effects of the applied stressors and treatment with extract on stress response were evaluated by measuring the stress hormone corticosterone in the serum of the experimental animals and glucocorticoid signalization in the hippocampus, one of the most important brain regions in response to stress. Hippocampal glucocorticoid signaling was investigated at the level of the enzymes involved in the prereceptor metabolism (11βHSD1 and H6PDH), which convert corticosterone locally in the tissue from the inactive to the active form, and at the level of the GR and its phosphorylation at Serine 211 and 226. Although concentration of corticosterone in the serum was not changed in female rats regardless of the treatment, the results showed a statistically significant effect of the extract on the level of H6PDH compared to control (** *p* < 0.01), the enzyme that provides the NADPH cofactor for 11βHSD1 (but also for other enzymes) while the protein level of 11βHSD1 was not changed (Figure 7). Chronic stress increased level of hippocampal GR in both female stressed groups regardless of the extract treatment (** *p* < 0.01, S vs. C; * *p* < 0.05 S + E vs. C) while the levels of its key phosphorylation sites pGR-Ser^226^, pGR-Ser^211^ and their ratio to total GR remained unchanged (Figure 7).

**Figure 8:**
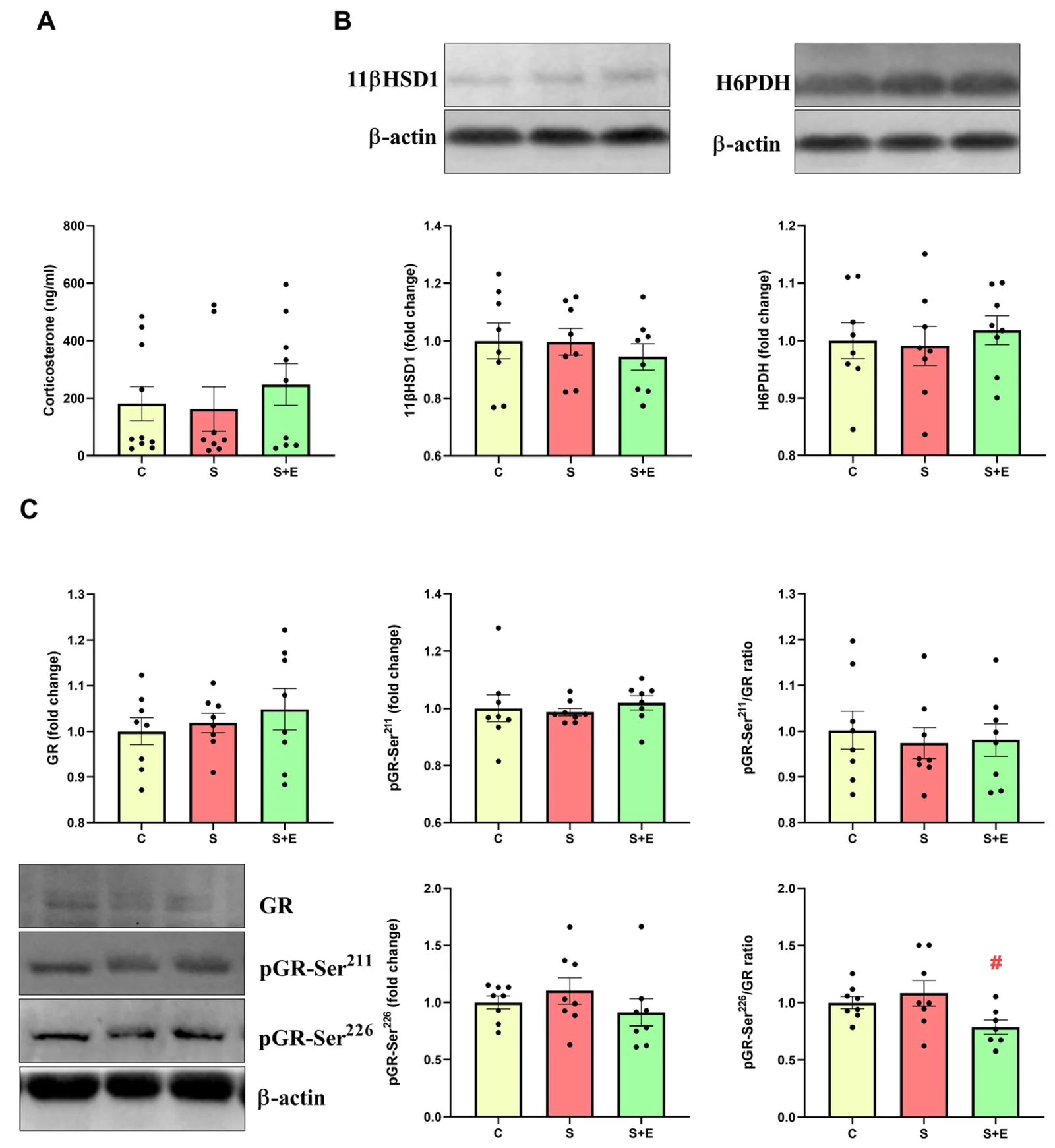
Glucocorticoid signaling pathway in the hippocampus of male rats. Corticosterone concentration (**A**), representative Western blots and protein levels of total 11βHSD1 and H6PDH (**B**), GR, pGR-Ser^226^, pGR-Ser^211^ and ratio of phosphorylated forms to total GR (**C**). The values represent the mean ± SEM (*n* = 7–9 animals per group) for each protein normalized to β-actin. Significant differences, between groups resulting from one-way ANOVA followed by Tukey’s post hoc test or non-parametric Kruskal–Wallis *H* test followed by Dunn’s post hoc test, are indicated as follows: ^#^ *p* < 0.05, S + E vs. S.

In males, ratio of pGR-Ser^226^ to GR was decreased in stressed rats treated with extract in comparison to stressed group (^#^ *p* < 0.05 S + E vs. S) (Figure 8). Other analyzed parameters of stress response were not changed in male rats regardless of the treatment (Figure 8).

### 2.8. Hippocampal Inflammation and Antioxidant Defense System

**Figure 9:**
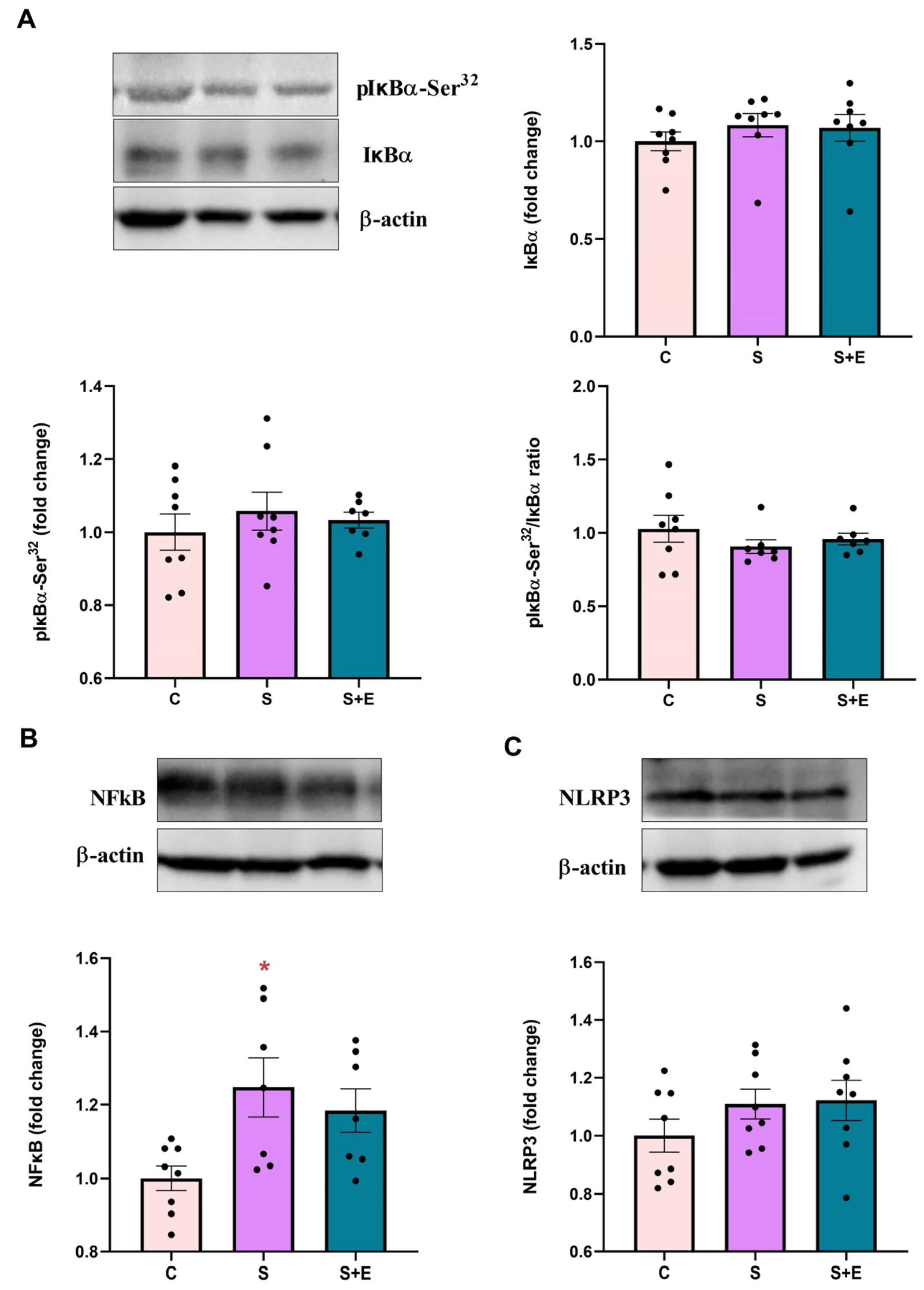
Inflammatory status in the hippocampus of female rats. Representative Western blots and protein levels of total IκBα, pIκBα-Ser^32^ and its ratio (**A**) NFκB (**B**) and NLRP3 (**C**). The values represent the mean ± SEM (*n* = 7–9 animals per group) for each protein normalized to β-actin. Significant differences, between groups resulting from one-way ANOVA followed by Tukey’s post hoc test or non-parametric Kruskal–Wallis *H* test followed by Dunn’s post hoc test, are indicated as follows: * *p* < 0.05, S vs. C.

Chronic stress exposure can change inflammatory status in different peripheral organs and brain regions. Therefore, we analyzed the effects of stress and extract on the inflammation in the hippocampus, by measuring hippocampal protein levels of NLRP3, as crucial part of innate immune system that forms an inflammasome in response to various stimuli, protein level of main proinflammatory transcriptional regulator NFkB, its inhibitory protein IkB and pIkB-Ser^32^. Our results showed that chronic stress increased protein level of NFkB (* *p* < 0.05, S vs. C) only in female stressed group of rats while extract treatment returned the values to the control (Figure 9B).

**Figure 10:**
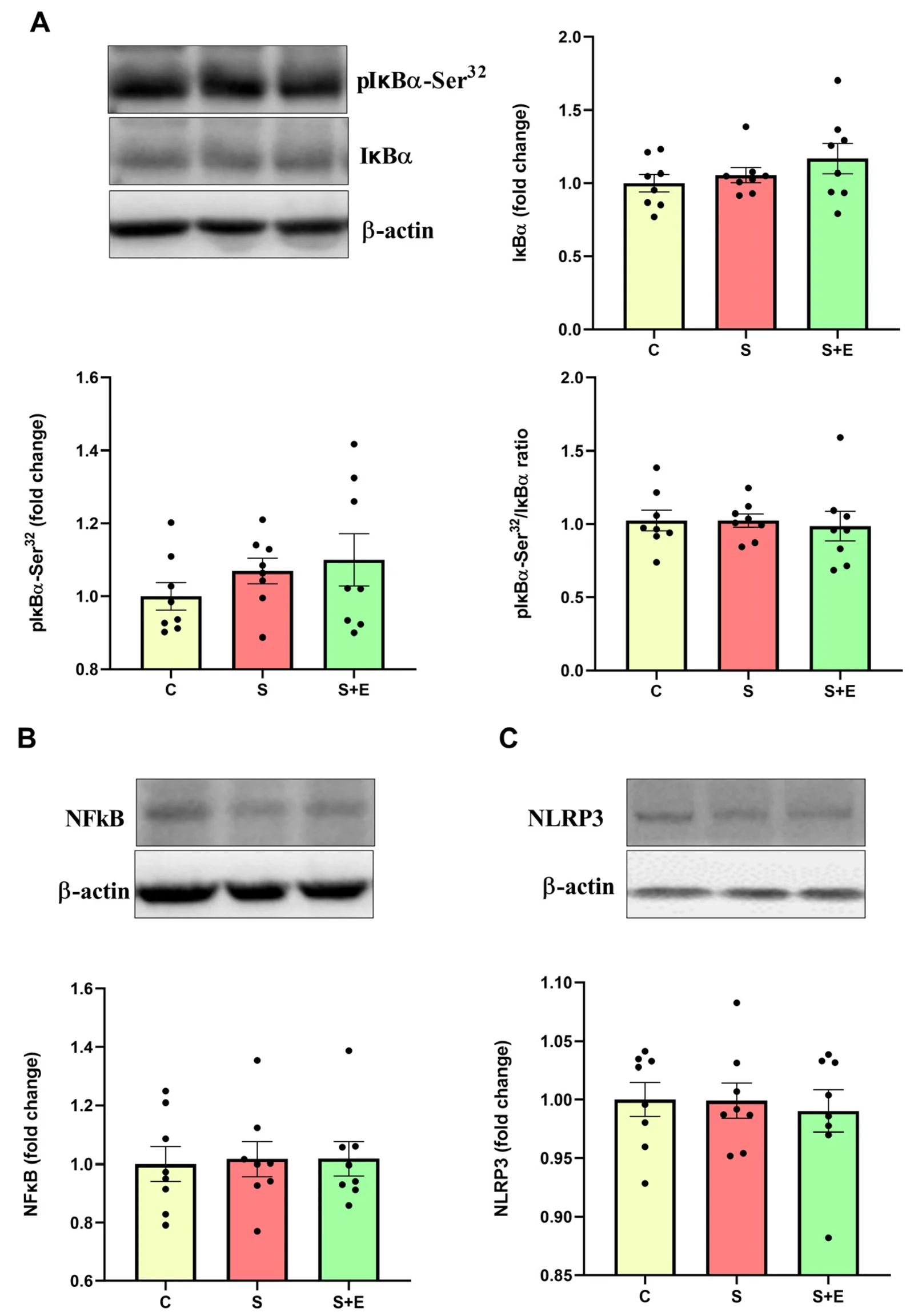
Inflammatory status in the hippocampus of male rats. Representative Western blots and protein levels of total IκBα, pIκBα-Ser^32^ and its ratio (**A**) NFκB (**B**) and NLRP3 (**C**). The values represent the mean ± SEM (*n* = 7–9 animals per group) for each protein normalized to β-actin.

Protein levels of NLRP3, IkB and pIkB-Ser^32^ remain unaltered in both males and females (Figure 9C and Figure 10).

**Figure 11:**
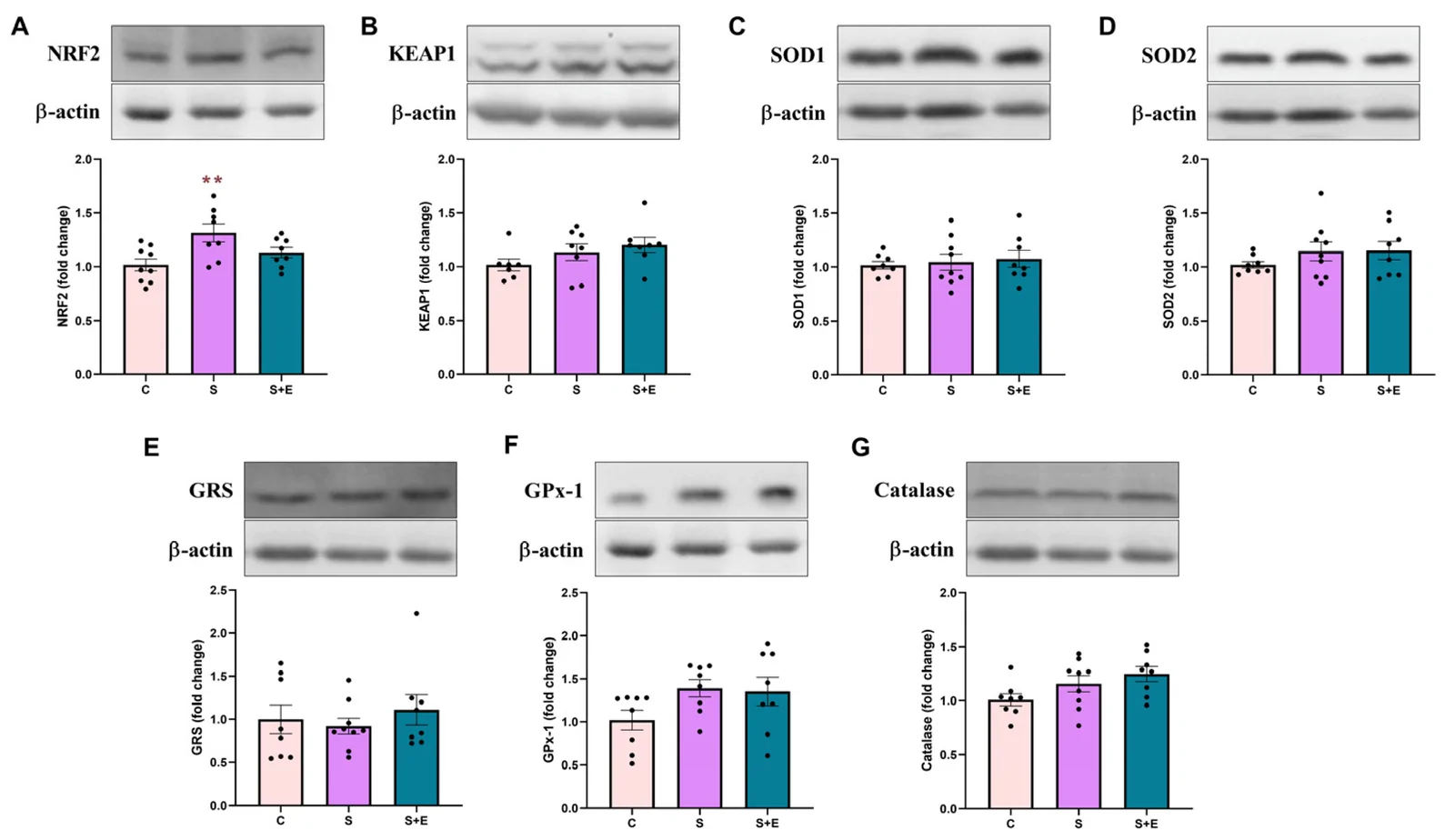
Anti-oxidative status in the hippocampus of female rats. Representative Western blots and protein levels of total NRF2 (**A**), KEAP1 (**B**), SOD1 (**C**). SOD2 (**D**), GRS (**E**), GPx-1 (**F**) and catalase (**G**). The values represent the mean ± SEM (*n* = 7–9 animals per group) for each protein normalized to β-actin. Significant differences, between groups resulting from one-way ANOVA followed by Tukey’s post hoc test or non-parametric Kruskal–Wallis *H* test followed by Dunn’s post hoc test, are indicated as follows: ** *p* < 0.01, S vs. C.

**Figure 12:**
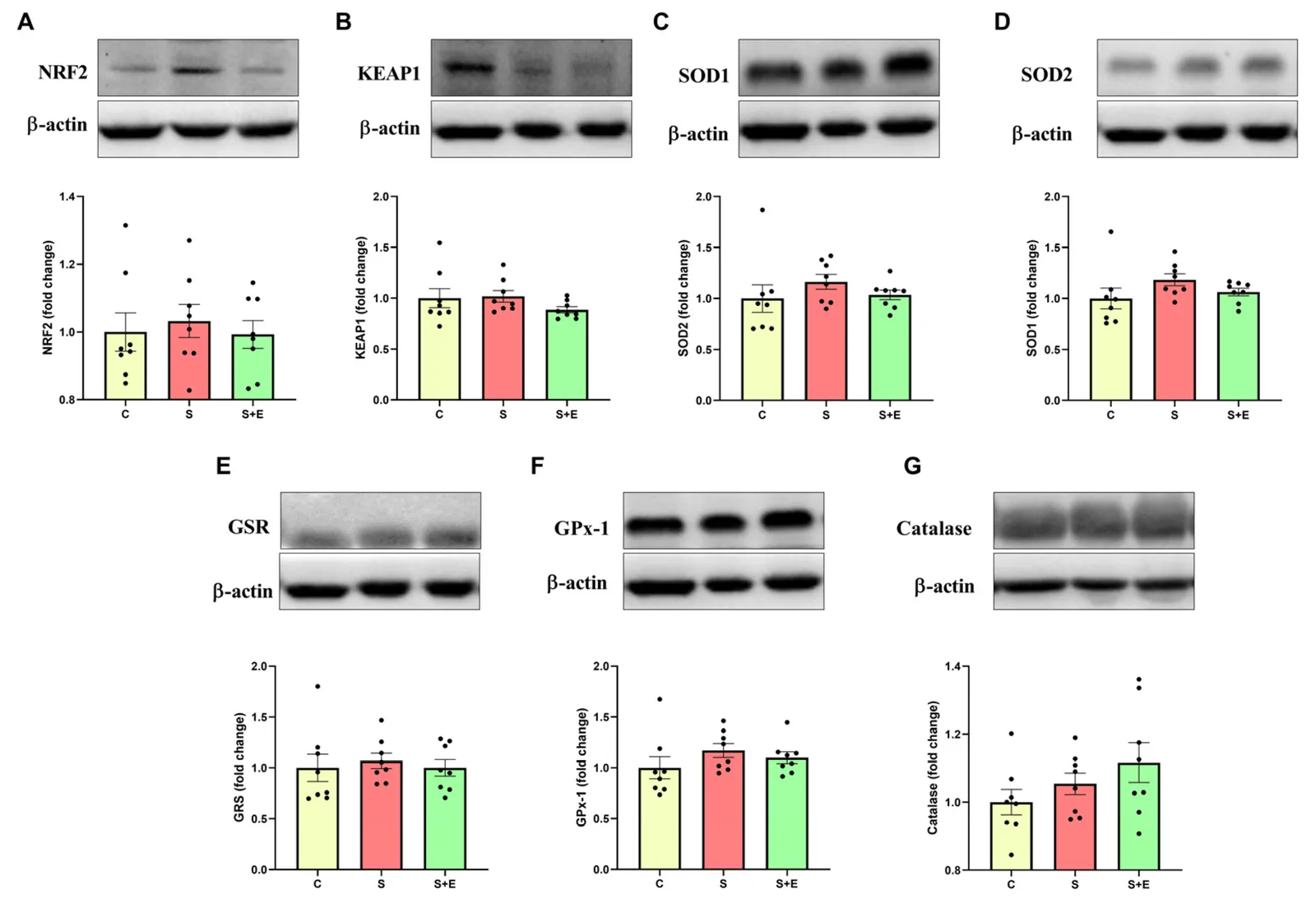
Anti-oxidative status in the hippocampus of male rats. Representative Western blots and protein levels of total NRF2 (**A**), KEAP1 (**B**), SOD1 (**C**). SOD2 (**D**), GRS (**E**), GPx-1 (**F**) and catalase (**G**). The values represent the mean ± SEM (*n* = 7–9 animals per group) for each protein normalized to β-actin.

Inflammatory status and antioxidant defense system of the hippocampus was assessed using protein concentrations of master transcriptional regulator of anti-oxidative defense NRF2, its negative regulator KEAP1 and antioxidant enzymes CAT, GRS, GPx-1, SOD1 and SOD2. There were no significant changes in the protein levels of CAT, GRS, GPx-1, SOD1 and SOD2 by either of the treatment applied in both, males and females (Figure 11 and Figure 12). Although the protein level of KEAP1 was not altered, the cellular concentration of NRF2 protein was increased only in female stress group of rats compared to the control group (** *p* < 0.01) while addition of extract returned its level to the one of unstressed rats (Figure 11 and Figure 12).

## 3. Discussion

In this study, chronic unpredictable stress induced pronounced metabolic, molecular, and behavioral alterations that were predominantly observed in female rats, whereas responses in males were comparatively modest. In females, chronic stress was associated with reduced body mass, altered glucose and insulin homeostasis, enhanced hippocampal glucocorticoid signaling, increased levels of key regulators of inflammatory and redox-sensitive signaling, and altered approach-avoidance behavior. Treatment with the herbal extract mixture containing *Centaurium erythraea*, *Valeriana officinalis*, *Melissa officinalis*, and *Humulus lupulus* attenuated several of these stress-associated alterations. Collectively, these findings indicate that, under the experimental conditions employed, the effects of chronic unpredictable stress and the response to extract treatment were predominantly detected in female rats.

The identified phytochemical profile provides a favorable chemical basis for the biological effects observed in the chronic unpredictable stress model, as the extract contained numerous phenolic acids, flavonoids, iridoids, and xanthones, compound classes previously associated with anti-inflammatory, neuroprotective, metabolic, and redox-modulating activities [24,25,26,27,28,29,30,31,32,33]. Among the identified constituents, rosmarinic acid, caffeoylquinic acids, quercetin derivatives, and luteolin have been reported to modulate NFκB- and NRF2-dependent signaling, improve glucose metabolism, and preserve hippocampal insulin signaling in experimental models [24,26,27,28,29]. These reported activities are consistent with the normalization of NFκB, NRF2, and IRS1 signaling in females observed in the present study. Because the formulation represents a complex phytochemical mixture, the biological effects are more likely attributable to complementary or additive interactions among multiple bioactive constituents than to the action of any single compound.

We found that chronic unpredictable stress, irrespective of extract treatment, reduced body mass in female but not male rats. Interestingly, food and energy intake remained unchanged in females, suggesting that the reduction in body mass was most likely driven by increased energy expenditure rather than decreased caloric intake. Previous studies have reported conflicting effects of chronic stress on body mass and food intake, ranging from hyperphagia and obesity to reduced appetite and weight loss [34,35]. These discrepancies are likely attributable to differences in type and duration of the stress exposure, animal strain, age and sex. Notably, most studies in female rodents have reported reduced body mass following chronic mild stress, indicating that females are particularly prone to stress-induced weight loss and hyperactivity [36,37]. These findings are consistent with our previous work, in which female rats exposed to the same chronic unpredictable stress protocol exhibited reduced body weight despite unchanged energy intake and hypothalamic expression of orexigenic and anorexigenic neuropeptides [38]. Given the well-established lipolytic actions of glucocorticoids on adipose tissue [39], the observed reduction in body mass is likely attributable, at least in part, to enhanced glucocorticoid-mediated lipolysis, as we have previously suggested in this model [40]. Treatment with herbal extract did not prevent this stress-induced weight loss.

To evaluate the effects of the herbal extract on the glucocorticoid response to chronic stress, we analyzed serum corticosterone levels together with key components of the hippocampal glucocorticoid signaling pathway, as the hippocampus is the one of brain regions most sensitive to stress and critically involved in the regulation of behavior. After five weeks of unpredictable stress, basal serum corticosterone levels remained unchanged in both sexes, irrespective of extract treatment, indicating that the applied stress protocol did not affect basal glucocorticoid levels. However, unchanged basal corticosterone does not necessarily reflect the overall glucocorticoid exposure during chronic stress. Indeed, our previous study, using the same stress protocol, as well as other studies, have shown that each stress episode elicits a transient corticosterone surge, resulting in repeated elevations of circulating glucocorticoids over the course of the stress protocol despite normal basal hormone concentrations [41,42]. Importantly, glucocorticoid action at the tissue level is determined not only by circulating hormone concentrations but also by prereceptor glucocorticoid metabolism and GR signaling. In females, chronic stress increased hippocampal GR levels in both stressed groups, while the level of the prereceptor enzyme H6PDH was increased in the hippocampus of stressed females receiving the herbal extract. In contrast, in males, the extract reduced the ratio of phosphorylated GR at Ser226 to total GR in stressed animals compared with untreated stressed controls, indicating decreased GR transcriptional activity and enhanced receptor nuclear export. Since there were no other changes in the hippocampal glucocorticoid signaling pathway in males, these findings suggest that male rats exhibited a relatively modest glucocorticoid response to chronic stress and that the extract may have contributed to maintaining glucocorticoid signaling homeostasis.

Chronic stress and prolonged glucocorticoid exposure have been associated with the development of both systemic insulin resistance and impaired hippocampal insulin signaling [6,43]. Interestingly, unlike males, female rats exhibited lower serum insulin and glucose concentrations following chronic stress exposure, with statistical significance observed in stressed and stressed group treated with extract, respectively. Consistent with these findings, the glucose AUC during the IP-GTT was reduced in stressed group, while the HOMA index was decreased in both female groups exposed to stress. Although reduced serum glucose levels and lower glucose AUC could initially be interpreted as improved glucose handling, their occurrence together with reduced circulating insulin suggests an altered metabolic adaptation to chronic stress rather than enhanced insulin sensitivity. In support of this interpretation, previous studies have demonstrated that chronic stress can reduce circulating insulin levels in several experimental models, including chronic unpredictable stress in rats [44], repeated immobilization stress [45] and chronic unpredictable environmental stress in mice [46]. However, many of these studies also reported hyperglycemia and impaired glucose tolerance, indicating the development of insulin resistance after chronic stress exposure. Recent evidence suggests that stress-induced alterations in glucose homeostasis represent a dynamic endocrine adaptation rather than a unidirectional progression toward insulin resistance [47]. Therefore, the reduced serum insulin observed in our study may represent an adaptive response to lower circulating glucose concentrations rather than impaired pancreatic β-cell dysfunction. Furthermore, reduced body mass, which is generally associated with improved insulin sensitivity, may also have contributed to the observed metabolic phenotype in stressed female rats.

Treatment with the herbal extract mixture appeared to attenuate stress-induced alterations of glucose homeostasis. During the IPGTT, chronic stress significantly decreased blood glucose concentrations at 15 min and 30 min compared to control females, whereas extract treatment restored glucose levels to values comparable to those of unstressed animals. Similar adaptive changes were observed in the hippocampus. Chronic stress increased total IRS1 protein level in the hippocampus of female rats, while extract treatment normalized IRS1 abundance toward control values and reduced the inhibitory phosphorylation of IRS1 at Ser307 compared with untreated stressed females. The increase in total IRS1 may represent a compensatory response to reduced circulating insulin, aimed at maintaining insulin responsiveness in the hippocampus. Consistent with this interpretation, no changes were detected in downstream insulin signaling components, including AKT, phosphorylated AKT, and GLUT4, suggesting that chronic stress did not induce hippocampal insulin resistance under the present experimental conditions. By restoring total IRS1 and pIRS1-Ser^307^ to control levels, the extract may reduce stress kinase-mediated inhibitory signaling and thereby stabilize hippocampal insulin signal transduction. Notably, these beneficial effects occurred despite persistently elevated hippocampal GR levels in stressed females treated with the herbal extract, suggesting that the extract acts downstream of GR activation and is more likely mediated by modulation of downstream stress pathways, possibly through interaction with redox-sensitive and inflammatory signaling mechanisms, rather than by a direct GR effect.

Inflammation and redox-sensitive signaling are key mechanisms linking chronic stress with metabolic dysfunction, including impaired insulin signaling. Therefore, we evaluated key regulators of inflammatory and redox-sensitive signaling in the hippocampus. No significant changes were observed in male rats, further supporting the relatively mild effects of the applied stress protocol in this sex. In contrast, female rats exhibited increased level of both NRF2 and NFkB following chronic stress. The increase in total NRF2 protein may represent an adaptive or compensatory response to an altered cellular redox environment. However, none of the examined downstream antioxidant proteins, including CAT, GRS, GPx-1, SOD1, and SOD2, showed significant changes. Thus, the increase in total NRF2 did not translate into detectable upregulation of these antioxidant proteins under the present experimental conditions. Importantly, total NRF2 abundance alone does not provide direct evidence of NRF2 transcriptional activity, which depends on several additional regulatory processes, including NRF2 stabilization, subcellular localization, nuclear translocation, and subsequent interaction with antioxidant response elements. Therefore, the present findings are more appropriately interpreted as alterations in NRF2-associated redox-sensitive signaling rather than as evidence of increased NRF2 transcriptional activity. Interestingly, treatment with the herbal extract normalized hippocampal levels of both NRF2 and NFκB. Rather than indicating suppression of endogenous antioxidant defenses, normalization of NRF2 may reflect a reduced redox challenge and consequently a diminished requirement for activation of cytoprotective pathways. This interpretation is consistent with the concurrent normalization of NFκB and supports the notion that the extract modulated stress-induced activation of redox-sensitive and inflammatory signaling pathways. This interpretation is supported by previous studies demonstrating anti-inflammatory and redox-modulating activities of all four plant species included in the extract mixture. *Humulus lupulus* and *Valeriana officinalis* extracts have been shown to inhibit NFκB signaling and decrease the level of pro-inflammatory cytokines [48,49], while *Melissa officinalis* extracts and newly isolated compounds from *Valeriana officinalis* exhibited high anti-inflammatory capacity [50,51]. It has also been reported that *Centaurium erythraea* has anti-inflammatory and antioxidant effects and that its extract can increase NRF2 and consequently SOD, CAT and GRS in neural tissue and peripheral organs in mice [49,52]. Interestingly, a recent study reported that *Humulus lupulus* administration did not alter hippocampal NRF2 expression and even reduced GPx and GRS activity in the brain [49]. As suggested by the authors, this may reflect a reduced requirement for endogenous antioxidant defense due to an improved redox environment, which could also be an explanation for our findings. The potential redox-modulating properties of the herbal mixture are consistent with previous studies showing that its individual components can scavenge reactive oxygen species and free radicals, reduce lipid peroxidation, and suppress the production of pro-oxidants [49,51,52,53,54,55], providing a plausible biological context for the effects observed in the present study. Accordingly, the redox-modulating properties of the extract may have contributed to the normalization of stress-associated redox-sensitive and inflammatory signaling, as reflected by the normalization of hippocampal NRF2 and NFκB levels. However, since the levels of the examined downstream antioxidant enzymes remained unchanged and direct markers of oxidative stress were not assessed, these findings should be interpreted as modulation of redox-sensitive signaling rather than as direct evidence of reduced oxidative stress. Given that redox-sensitive and inflammatory signaling pathways can promote inhibitory IRS1 serine phosphorylation, modulation of these pathways provides a plausible link between extract treatment and preservation of hippocampal insulin signaling.

The molecular changes observed in the hippocampus were accompanied by behavioral alterations that were predominantly detected in female rats. Chronic stress was associated with altered approach-avoidance behavior in female rats, as evidenced by a significant reduction in the time spent in the center zone of the OFT, whereas center-zone time in extract-treated stressed females did not differ significantly from either the control or stressed group. No significant behavioral changes were detected in males. Interestingly, these behavioral effects became evident only during the later phase of the OFT. During the first 20 min, stressed and control females displayed similar exploratory behavior, whereas later both stressed groups exhibited reduced locomotor and stereotypic activity together with less time spent in the central zone. This temporal pattern indicates that the effects of chronic stress were not evident during the initial exposure to the novel environment but emerged during prolonged exploration of the arena.

These findings are particularly interesting considering the composition of the herbal mixture. Two of its components, *Melissa officinalis* and *Valeriana officinalis*, have well-documented anxiolytic properties demonstrated in both experimental and clinical studies [13,18,56]. Based on these properties, we expected the four-plant mixture to produce a detectable anxiolytic-like behavioral profile and therefore included the EPM, a well-established and widely validated paradigm for assessing anxiety-related behavior [57]. However, the EPM did not reveal significant differences among the experimental groups and thus did not provide direct evidence of an anxiolytic effect of the extract.

Interestingly, the OFT revealed a more nuanced behavioral pattern. Although a significant direct comparison between stressed and extract-treated stressed animals was not observed for total center-zone time, extract-treated stressed females did not differ significantly from controls in this measure. Together with the temporal pattern observed in the OFT, these findings raise the possibility that the extract may modulate the approach-avoidance component of exploratory behavior under conditions of chronic stress. In the OFT, center exploration reflects the balance between the motivation to explore a novel environment and avoidance of an exposed and potentially aversive area [58]. Reduced center exploration is commonly interpreted as an anxiety-related behavioral measure, as animals exhibiting greater avoidance of the exposed central area tend to spend more time in the peripheral zone and display increased thigmotaxis [59]. Thus, the present findings may be better interpreted as modulation of a specific component of the stress-related behavioral response rather than as evidence of a generalized anxiolytic effect. The lack of a corresponding effect in the EPM further indicates that this behavioral modulation may be context-dependent, as the two paradigms involve different environmental demands and aversive stimuli [60].

The observation that behavioral alterations were detected predominantly in female rats is also of interest. Previous studies have reported differences between females and males in behavioral responses to chronic stress [36,61,62], whereas the anxiolytic properties of *Melissa officinalis* and *Valeriana officinalis* have been reported in both sexes [13,18,56]. In the present study, however, no formal sex × group interaction was tested and therefore, the differences in statistical significance observed between females and males should not be interpreted as evidence of a sex-specific effect of the extract. Further studies specifically designed to examine sex-related differences in behavioral responses to the polyherbal mixture are warranted.

The behavioral observations may also be considered in relation to the molecular changes detected in the hippocampus. The behavioral effects of *Melissa officinalis* in both male and female rats have previously been associated with reduced oxidative stress in the brain [18]. Although oxidative stress was not directly assessed in the present study, this observation raises the possibility that modulation of redox-sensitive signaling may contribute to the behavioral responses associated with the herbal mixture. In addition, disturbances in whole-brain and hippocampal insulin signaling have been associated with alterations in behavior, mood, memory, and anxiety-related behavior [63,64]. Therefore, the normalization of hippocampal NFκB, NRF2, and IRS1 signaling observed in the present study may represent molecular changes potentially associated with the observed behavioral profile following extract treatment.

Taken together, these findings should be considered exploratory and do not establish a definitive anxiolytic effect of the extract. Nevertheless, the behavioral pattern observed in the OFT, together with the absence of corresponding effects in the EPM and the concomitant changes in hippocampal molecular signaling, raises the possibility that the herbal mixture may modulate specific components of the behavioral response to chronic stress, particularly those related to approach-avoidance behavior. Under the experimental conditions employed, the behavioral, molecular, and metabolic alterations were predominantly detected in female rats, whereas responses in males were comparatively modest. This limits the extent to which the effects of the extract can be evaluated across both sexes; moreover, formal conclusions regarding sex-related differences cannot be drawn from the present analyses, representing a limitation of the study. Further studies using additional behavioral paradigms, different doses, and experimental designs specifically powered to assess sex-related effects are warranted.

Several additional limitations should also be considered. Redox-related alterations were assessed through redox-sensitive signaling pathways rather than by direct measurement of ROS or oxidative damage; therefore, the present findings do not establish a direct effect of the extract on oxidative stress. Finally, the observed effects can be attributed only to the polyherbal formulation as a whole, while the relative contributions of individual phytochemical constituents and their potential additive or synergistic interactions remain to be established.

In conclusion, the polyherbal extract containing *Centaurium erythraea*, *Valeriana officinalis*, *Melissa officinalis*, and *Humulus lupulus* attenuated several metabolic and molecular alterations associated with chronic unpredictable stress, with these effects predominantly observed in female rats under the experimental conditions employed. The effects of the extract were associated with improved glucose homeostasis and normalization of hippocampal NFκB, NRF2, and IRS1 levels, suggesting modulation of stress-associated inflammatory, redox-sensitive, and insulin signaling pathways downstream of GR activation. Behavioral findings were more limited and did not provide evidence of a generalized anxiolytic effect. Together, these findings suggest that modulation of inflammatory, redox-sensitive, and insulin signaling pathways downstream of GR activation may contribute to the protective metabolic and molecular effects of the polyherbal extract during chronic stress. Further studies are required to establish its behavioral effects and determine whether its actions differ between females and males.

## 4. Materials and Methods

### 4.1. Plant Material and Extract Preparation

Dried plant material was obtained from the commercial supplier, the Institute for Medicinal Plant Research “Dr Josif Pančić” (Belgrade, Serbia). The herbal mixture consisted of 40 g of lemon balm leaves (*Melissa officinalis* L., *Lamiaceae*), 20 g of valerian root and rhizome (*Valeriana officinalis* L., *Caprifoliaceae*), 20 g of hop strobiles (*Humulus lupulus* L., *Cannabaceae*), and 20 g of common centaury herb (*Centaurium erythraea Rafn*, *Gentianaceae*), yielding a total of 100 g of dried plant material. The mixture was transferred into an Erlenmeyer flask, and 1000 mL of 70% (*v*/*v*) ethanol was added as the extraction solvent. Ultrasound-assisted extraction was performed in an ultrasonic bath for 30 min at 25 °C and 100% bath power (35 W amplitude). Following sonication, the extraction mixture was kept at 4 °C for 24 h to facilitate further diffusion of phytochemicals into the solvent. Subsequently, the flask was returned to the ultrasonic bath and subjected to a second sonication step for 30 min at 25 °C using 90% bath power (35 W amplitude). The extract was then filtered, and the filtrate was stored at 4 °C until all extraction cycles had been completed. The remaining plant residue was re-extracted under identical ultrasonic conditions using 715 mL of fresh 70% (*v*/*v*) ethanol. This two-step extraction procedure, consisting of sonication, cold maceration, re-sonication, filtration, and re-extraction of the plant residue, was repeated four independent times, resulting in the extraction of a total of 400 g of the herbal mixture. Upon completion of all extraction cycles, the individual filtrates were pooled, filtered once more to remove residual particulate matter, and concentrated under reduced pressure using a rotary evaporator (Büchi R-210, Flawil, Switzerland). Solvent removal yielded a dark brown, highly viscous crude extract, which was transferred into an appropriate container and stored for subsequent analyses. The overall extraction yield was 49.0% (*w*/*w*), calculated relative to the initial dry weight of the plant material. For phytochemical characterization, an aliquot of the concentrated crude extract was dissolved in HPLC-grade methanol (99.9%) to obtain a final concentration of 1 mg/mL. The resulting solution was subjected to ultra-high-performance liquid chromatography coupled with Orbitrap high-resolution mass spectrometry (UHPLC-Orbitrap HRMS, Thermo Scientific™, Waltham, MA, USA) for comprehensive metabolite profiling.

### 4.2. Chemical Characterization of Polyherbal Mixture Extract

The extract’s metabolic profile was ascertained using the Liquid Chromatography–High-Resolution Tandem Mass Spectrometry (LC-HRMS/MS, Thermo Scientific™ Vanquish™ Core HPLC system, Thermo Scientific™, Waltham, MA, USA) connected to the Orbitrap Exploris 120 mass spectrometer, San Jose, CA, USA. The Hypersil GOLDTM C18 analytical column (50 × 2.1 mm, 1.9 μm particle size) was part of the liquid chromatography system [65]. The flow rate remained steady at 300 μL/min, and the injection volume was 5 μL. Ultrapure water + 0.1% formic acid (A) and acetonitrile (MS grade) + 0.1% formic acid (B) were used to elute the compounds of interest: 5% B for the first min, 5–95% B for 1–10 min, 95% B for 10–12 min, and 5% B for 15 min.

An ESI source running in negative ionization mode was installed in the Orbitrap Exploris 120 mass spectrometer (Thermo Scientific™, Waltham, MA, USA). While data-dependent MS2 tests were carried out at an Orbitrap resolution of 15,000 FWHM with a normalized collision energy of CID set to 35%, full-scan MS was monitored from 100 to 1500 *m*/*z* with an Orbitrap resolution of 60,000 FWHM. The intensity threshold was set to 1 × 10^5^, and the dynamic exclusion duration was set to 10 s with exclusion from a particular scan after two occurrences. Based on their chromatographic behavior, MS and MS2 comparisons with standard compounds, when available, and literature data that offered a preliminary identification, the compounds were identified. Data acquisition was carried out with Xcalibur^®^ data system (Thermo Finnigan, San Jose, CA, USA).

### 4.3. In Vitro Biosafety Assay on Human Keratinocytes

The in vitro biosafety of the polyherbal extract was evaluated using the crystal violet assay with slight modifications of previously described protocol [66]. Immortalized human keratinocytes (HaCaT; AdexxBio, T0020001) were used as a non-tumorigenic cell model to assess the cytotoxic potential of the extract. HaCaT cells were cultured in Dulbecco’s Modified Eagle Medium (DMEM) supplemented with 10% fetal bovine serum (FBS), 100 U/mL penicillin, and 100 μg/mL streptomycin under standard culture conditions (37 °C, 5% CO_2_, and humidified atmosphere). Cells were seeded into 96-well plates at a density of 4 × 10^3^ cells per well and allowed to adhere for 48 h. The polyherbal extract was initially dissolved in culture medium to obtain a stock solution of 8 mg/mL, from which working solutions were freshly prepared by serial dilution. Cells were exposed to final extract concentrations of 6.25, 12.5, 25, 50, 100, 200, and 400 μg/mL. Untreated cells cultured in complete DMEM served as negative control. The experiment was performed independently three times. In each experiment, each treatment was tested in triplicate wells, which served as technical replicates. The values obtained from the technical replicates were averaged for each independent experiment, and these means were used for subsequent statistical analysis (*n* = 3 independent experiments). Cells were incubated with the extract for 24 and 48 h.

Following treatment, the culture medium was removed, and the cells were washed twice with phosphate-buffered saline (PBS). Subsequently, 100 μL of 0.4% (*w*/*v*) crystal violet solution was added to each well, and the plates were incubated at room temperature for 20 min. Excess stain was removed by washing under running tap water, after which the plates were air-dried for 24 h. The bound dye was solubilized with 100 μL of methanol per well, and absorbance was measured at 570 nm using a microplate reader. Cell viability was expressed as the relative growth rate (%) compared with untreated control cells after 24 and 48 h of exposure. The biosafety of the extract was evaluated according to the criteria defined in ISO 10993-5 [67] for in vitro cytotoxicity testing. Samples maintaining ≥70% cell viability relative to untreated control cells were considered non-cytotoxic and therefore biosafe under the tested experimental conditions.

### 4.4. Animals and Treatment

Two-and-a-half-month-old male and female Wistar rats bred in the animal facility of the Institute for Biological Research “Siniša Stanković” were randomly allocated to cages and three experimental groups at the beginning of the study (*n* = 7–9 animals per group), the untreated control group (C), the stress group (S) exposed to chronic unpredictable stress for 5 weeks, and the stress + herbal extract mixture group (S + E) exposed to the same stress protocol as the S group for 5 weeks, with the addition that during the last 3 weeks of stress these animals received a polyherbal extract administered orally by gavage (300 mg/kg). No a priori inclusion or exclusion criteria were established. Oral gavage was performed on the rats from C and S groups as well, but they were receiving tap water instead of plant extract. According to OECD guideline for testing of chemicals, acute toxicity test was performed using three doses (50, 300 and 2000 mg/kg) of herbal extract mixture administrated orally to three female rats per dose. No treatment-related signs of toxicity or mortality were observed at any of the tested doses. The chronic unpredictable stress protocol, adapted from [3], involved a randomized schedule of daily stressors, including physical restraint for 60 min, forced swimming in cold water for 10 min, cage rocking for 1 h, cage switching for 2 h, and overnight cage tilting at a 45° angle. The stressors were applied once or twice daily at randomized times (between 9:00 and 16:00, with the exception of overnight tilting), ensuring that no single stressor was repeated on consecutive days.

The animals were housed in groups of three per cage under standard conditions (22 °C, 12 h light/dark cycle with lights on at 7:00 A.M.). All rats had ad libitum access to drinking water and commercial rat chow [Laboratory Rat Food R20: 62.6% carbohydrates, 20% protein and 3.2% fat with vitamins and minerals; Veterinary Institute Subotica, Serbia]. Food and fluid intake were measured daily, while body mass was recorded weekly. Food, fluid and energy intake were expressed as the average weekly intake per body mass of the animals. Energy intake was calculated by summing the energy consumed in the diet (mass of the food ingested per day per cage (g) × 11 kJ). Animal husbandry and routine management were performed by trained animal facility staff according to standard institutional procedures. Animals were regularly monitored throughout the experiment by animal facility staff under veterinary supervision. No predefined humane endpoints were established, and no animals developed clinical signs requiring early euthanasia. Different researchers were responsible for animal experimentation, biochemical and molecular analyses, and data analysis; formal blinding was not applied. The study was conducted in accordance with the ethical regulations of the European Commission for Animal Protection (2010/63/EU) and was approved by the Ethics Committee for the Use of Laboratory Animals of the Ministry of Agriculture, Forestry and Water Management of the Republic of Serbia (decision number 323-07-10170/2023-05 dated 18 September 2023).

### 4.5. Behavioral Testing

Male and female rats were subjected to the OFT and three days later to the EPM test, between 9:00 and 17:00 in a room under consistent conditions. The OFT and EPM apparatuses were thoroughly cleaned with a 10% alcohol solution and dried, and the room was ventilated to remove odors between sessions. Freely behaving animals were recorded using a light-sensitive camera (Logitech C270 HD-960-001063, Lausanne, Switzerland) mounted 1.5 m above the OFT and EPM apparatuses. All recordings were securely stored on a hard disk until further behavioral analysis using ANY-maze video tracking software (version 7.2, Stoelting Co., Wood Dale, IL, USA).

#### 4.5.1. Open Field Test

The OFT arena was made of white opaque Plexiglas with a light-gray bottom and measured 41 × 41 × 41 cm. The setup consisted of 4 arenas and allowed simultaneous recording of 4 animals. Animals were placed in one corner of the OF arena and allowed to explore it for 60 min. The following behaviors were monitored: locomotor activity expressed as distance traveled (m), stereotypy-like activity expressed as head distance traveled (m), and anxiety-like behavior expressed as time in the center zone (s). To score entries and exits in the center zone, which is half the size of the arena, at least 70% of the animal must be in the zone for an entry to occur, and at least 50% of the animal must remain in the zone for an exit not to occur.

#### 4.5.2. Elevated Plus Maze

The EPM apparatus was made of blue acrylic and consisted of two opposite open arms (50 × 10 cm) and two opposite closed arms (50 × 10 × 40 cm) connected by a central platform (10 × 10 cm). The apparatus was elevated 50 cm above the floor. Animals were placed one by one on the central platform facing an open arm and recorded for 10 min. To assess anxiety-like behavior, the following parameters were monitored: time in the open and closed arm (s) and number of entries to the open and closed arm. To score entries and exits in the open and closed arm, at least 80% of the animal must be in the arm for an entry to occur, and at least 70% of the animal must remain in the arm for an exit not to occur. Higher activity in the open arms was an indicator of lower anxiety-like behavior.

### 4.6. Serum Preparation and Measurement of Biochemical Parameters

After fasting for 4 h, the animals were sacrificed by rapid decapitation, and trunk blood was collected consistently between 10:00 a.m. and 12:00 p.m. Blood was collected into tubes and allowed to clot at room temperature for 30 min. Serum was separated by centrifugation at 2000× *g* for 10 min and stored at −20 °C until further analysis.

Serum corticosterone levels (CORT) were measured using the Corticosterone EIA kit according to the manufacturer’s instructions (Immunodiagnostic Systems Ltd., Boldon, UK). Insulin concentration in serum was determined using the rat/mouse insulin ELISA kit (#EZRMI-13K) according to the manufacturer’s instructions (EMD Millipore Corporation, St. Louis, MO, USA). The sensitivity of the assay was 0.1 ng/mL, and the intra-assay coefficient of variation was 1.92%.

### 4.7. Quantification of Insulin Sensitivity Parameters

Systemic insulin sensitivity was evaluated through two distinct methods, the HOMA index and the IP-GTT. The HOMA index was determined based on fasting insulin and glucose levels and calculated using the following formula: [insulin (mIU/L) × glucose (mmol/L)]/22.5. IP-GTT was performed 3 days before the end of treatment. Rats were fasted for 4 h, and glucose was administered intraperitoneally (2 g/kg) without anesthesia to avoid the effect of the anesthetic on glucose levels and the kinetics of glucose utilization. The glucose concentration was determined from the blood in the tail vein at 0, 15, 30, 60, 90 and 120 min after injection. The glucose concentration in the blood was measured with AccuChek Active^®^ strips (F. Hoffmann-La Roche AG, Basel, Switzerland). The area under the concentration vs. time curve (AUC glucose 0–120 min, mmol/L vs. min) was calculated according to the trapezoidal rule.

### 4.8. Preparation of Hippocampal Whole Cell Extracts

Immediately after decapitation, the hippocampus was isolated on ice, frozen in liquid nitrogen and stored at −70 °C until use. To prepare whole cell extracts, tissue was homogenized using a Teflon glass homogenizer in ice-cold RIPA buffer (50 mM Tris HCl pH 7.2, 150 mM NaCl, 2 mM DTT, 1 mM EDTA Na2, 1% NP40, 0.5% sodium deoxycholate, 0.1% SDS, and phosphatase and protease inhibitors). The homogenates were sonicated (3 × 5 s, 1 A, 50/60 Hz), incubated on ice for 60 min with constant agitation and frequent vortexing, and the suspensions were centrifuged (16,000× *g*, 20 min, 4 °C). The resulting supernatants were stored at −70 °C and used as whole cell extracts.

### 4.9. Western Blot Analyses

Protein concentration was determined according to the Lowry method [23], and 40 µg of proteins were subjected to electrophoresis on 7.5% or 12% sodium dodecyl sulfate-polyacrylamide gels (SDS-PAGE). After transfer to a polyvinylidene difluoride (PVDF) membrane (Amersham™ Hybond^®^ P membrane, Sigma Aldrich, St. Louis, MO, USA) using a Transblot system (Bio-Rad Laboratories, Hercules, CA, USA), the unbound sites were blocked (1 h with 5% non-fat dry milk) and the membranes were incubated overnight at 4 °C with specific primary rabbit polyclonal antibodies for anti-GR (1:500) (sc-8992, Santa Cruz Biotechnology, Dallas, TX, USA), anti-11βHSD1 (1:1000) (ab393364, Abcam, Cambridge, UK), anti-H6PDH (1:1000) (sc-67394, Santa Cruz Biotechnology, Dallas, TX, USA), anti-Catalase (1:1000) (ab16731, Abcam, Cambridge, UK), anti-GRS (1:2000) (ab16801, Abcam, Cambridge, UK), anti-GPx (1:2000) (ab22604, Abcam, Cambridge, UK), anti-SOD1 (1:2000) (FNab08103, FineTest, Wuhan, China), anti-SOD2 (1:2000) (ab13533, Abcam, Cambridge, UK), anti-NRF2 (1:500) (FNab05855, FineTest, Wuhan, China), anti-KEAP1 (1:500) (FNab04516, FineTest, Wuhan, China), anti-IRS1 (1:1000) (FNab10261, FineTest, Wuhan, China), anti-phospho-IRS1 (Ser307) (1:500) (ab5599, Abcam, Cambridge, UK), anti-NFκB p65 (1:500) (8242, Cell Signaling Technology, Danvers, MA, USA), anti-IκBα (1:500) (924, Cell Signaling Technology, Danvers, MA, USA), anti-phospho-IκBα (Ser32) (1:500) (9241, Cell Signaling Technology, Danvers, MA, USA), anti-phospho-GR (Ser211) (1:500) (PA5-99335, Thermo Fisher Scientific, Waltham, MA, USA), anti-phospho-GR (Ser226) (1:500) (PA5-104735, Thermo Fisher Scientific, Waltham, MA, USA), and anti-NLRP3 (1:1000) (NBP2-12446, Novus Biologicals, Centennial, CO, USA). The anti-β-actin antibody (1:10,000) (ab8227, Abcam, Cambridge, UK) was used as a loading control. Blots were incubated for 90 min with an HRP-linked anti-rabbit IgG HRP-linked secondary antibody (1:20,000) (ab6721, Abcam, Cambridge, UK). The immunoreactive protein bands were detected by the chemiluminescent (ECL) method using iBright CL1500 (Thermo Fisher Scientific, Waltham, MA, USA), and quantitative analysis of protein bands was performed using Image J software 1.52a (NIH, Bethesda, MD, USA).

### 4.10. Statistical Analyses

The data are presented as mean values ± SEM. All data were tested for normality using the Shapiro–Wilk test. Unless otherwise stated, normally distributed data were analyzed using one-way ANOVA followed by Tukey’s post hoc test. Data with deviation from the normal distribution were analyzed using the non-parametric Kruskal–Wallis *H* test followed by Dunn’s post hoc test. The differences between the groups were considered significant at *p* < 0.05. Statistical analyzes were performed using GraphPad Prism 8 software (San Diego, CA, USA).

## 5. Patents

The polyherbal extract investigated in this study is the subject of a Serbian national patent application filed by the Institute for Biological Research “Siniša Stanković”, University of Belgrade (patent application no. P-2024/0534).

## Figures and Tables

**Table 1:** Selected abundant and representative metabolites tentatively identified in the polyherbal extract and their semi-quantitative contents.

Chemical Class	Compound	Semi-Quantitative Content (mg/kg) *
Hydroxybenzoic acid derivatives	Hydroxybenzoyl-galloyl hexoside	5058.69
Hydroxycinnamic acid derivatives	Sagerinic acid	17,774.56
Hydroxycinnamic acid derivatives	Rosmarinic acid	10,471.47
Hydroxycinnamic acid derivatives	Lithospermic acid	7511.84
Hydroxycinnamic acid derivatives	Schizotenuin A	6904.05
Iridoid glycosides	Caffeoyl-loganin	8113.97
Iridoid glycosides	Alpigenoside	5811.64
Iridoid glycosides	1-*O*-hexosyl-loganic acid (Asystasioside A)	2188.86
Flavonoid C-glycosides	Apigenin 8-*C*-hexoside	277.91
Flavonoid aglycones	Hispidulin	291.21
Xanthone glycosides	Trihydroxy-dimethoxy xanthone *O-*rhamnosyl-hexoside	166.41
Xanthone aglycones	Trihydroxy-methoxy xanthone (Swertianin)	83.1

* Semi-quantitative contents were estimated using calibration curves of representative reference compounds for the corresponding phytochemical classes: gallic acid for hydroxybenzoic acid derivatives, caffeic acid for hydroxycinnamic acid derivatives, loganin for iridoid glycosides, rutin for flavonoid glycosides, quercetin for flavonoid aglycones, and mangiferin for xanthone derivatives. Values are therefore expressed as equivalents of the respective class-specific reference compounds and should be interpreted as semi-quantitative estimates rather than absolute concentrations determined using authentic standards for individual metabolites.

**Table 2:** Food and energy intake and total body mass of female and male rats.

	C	S	S + E
	**Females**		
Food intake (g/day/animal)	22.20 ± 0.22	21.79 ± 1.40	20.16 ± 0.32
Energy intake (kJ/day/animal)	73.92 ± 0.75	72.55 ± 4.65	67.12 ± 1.06
Body mass (g)	314.11 ± 7.97	281.80 ± 4.94 **	290.33 ± 3.98 *
	**Males**		
Food intake (g/day/animal)	28.63 ± 1.42	27.81 ± 0.95	24.72 ± 1.00
Energy intake (kJ/day/animal)	100.46 ± 0.87	98.26 ± 4.51	86.96 ± 1.82 ^#,^*
Body mass (g)	449.10 ± 19.53	446.00 ± 18	402.3 ± 12.46

Data are given as mean values ± SEM (*n* = 7–9 animals per group). Significant differences between groups resulting from one-way ANOVA followed by Tukey’s post hoc test or non-parametric Kruskal–Wallis *H* test followed by Dunn’s post hoc test are indicated as follows: * *p* < 0.05, S + E vs. C group, ** *p* < 0.01, S vs. C group; ^#^ *p* < 0.05, S + E vs. S group.

## Data Availability

All data generated or analyzed during this study are included in this published article and its Supplementary Information files.

## Supplementary Materials

The following supporting information can be downloaded at https://www.mdpi.com/article/10.3390/ijms27188298/s1.

## Abbreviations

The following abbreviations are used in this manuscript:

AKT	Protein kinase B
AUC	Area under the curve
CAT	Catalase
DMEM	Dulbecco’s Modified Eagle Medium
ECL	Enhanced chemiluminescence
EDTA	Ethylenediaminetetraacetic acid
ELISA	Enzyme-linked immunosorbent assay
EPM	Elevated plus maze
ESI	Electrospray ionization
FBS	Fetal bovine serum
GLUT4	Glucose transporter type 4
GPx	Glutathione peroxidase
GR	Glucocorticoid receptor
GRS	Glutathione reductase
H6PDH	Hexose-6-phosphate dehydrogenase
HOMA	Homeostatic Model Assessment
HPA	Hypothalamic–pituitary–adrenal
11βHSD1	11β-Hydroxysteroid dehydrogenase type 1
IP-GTT	Intraperitoneal glucose tolerance test
IRS1	Insulin receptor substrate 1
KEAP1	Kelch-like ECH-associated protein 1
LC-HRMS/MS	Liquid chromatography–high-resolution tandem mass spectrometry
MS/MS	Tandem mass spectrometry
NADPH	Nicotinamide adenine dinucleotide phosphate (reduced form)
NFκB	Nuclear factor kappa B
NLRP3	NLR family pyrin domain-containing protein 3
NRF2	Nuclear factor erythroid 2-related factor 2
OA	Open arms
OFT	Open field test
PBS	Phosphate-buffered saline
PVDF	Polyvinylidene fluoride
ROS	Reactive oxygen species
SDS-PAGE	Sodium dodecyl sulfate–polyacrylamide gel electrophoresis
SEM	Standard error of the mean
SOD	Superoxide dismutase
UHPLC	Ultra-high-performance liquid chromatography
