# Supplementary material for: A Chemically Characterized Polyherbal Extract Selectively Attenuates Chronic Unpredictable Stress-Induced Redox-Sensitive, Inflammatory, and Metabolic Alterations in Female Rats
Source: Int J Mol Sci. 2026 Sep 17;27(18):8298. doi: 10.3390/ijms27188298 (PMC13607128; doi:10.3390/ijms27188298)
Supplement: Supplementary file 1 [file ijms-27-08298-s001.zip › Supplemenatary Table S1.pdf]

Table S1. Identification of metabolites in herbal tea made from a mixture of *Melissa officinalis* (Lamiaceae), *Humulus lupulus* (Cannabaceae), *Valeriana officinalis* (Caprifoliaceae), and *Centaurium erythraea* (Gentianaceae). Gentianaceae and Lamiaceae-specific compounds dominate the extract.

**No**	**Compound name**	t_R, **min**	**Molecular formula, [M–H]⁻**	**Calculated mass, [M–H]⁻**	**Exact mass, [M–H]⁻**	**Δ ppm**	**MS² Fragments, (% Base Peak)**
Hydroxybenzoic acid derivatives							
**1**	**Dihydroxybenzoyl hexoside**	0.49	$C_{13}H_{15}O_9^-$	315.07216	315.07225	-0.29	109.02956(53), **153.01944**(100), 153.05504(15), 315.07251(33)
**2**	**Galloyl hexoside**	0.55	$C_{13}H_{15}O_{10}^-$	331.06707	331.06721	-0.41	125.02479(59), 151.00378(80), 169.01469(61), **331.06522**(100)
**3**	**Gallic acid**	0.61	$C_7H_5O_5^-$	169.01425	169.01424	0.05	**125.02448**(100), 169.01427(37)
**4**	**Vanniloyl hexoside**	0.66	$C_{14}H_{17}O_9^-$	329.08781	329.08794	-0.40	**123.04529**(100), 167.03493(15)
**5**	**Hydroxybenzoyl hexoside**	0.74	$C_{13}H_{15}O_8^-$	299.07724	299.07738	-0.45	93.03465(16), **137.02452**(100)
**6**	**Dihydroxybenzoyl hexoside**	0.91	$C_{13}H_{15}O_9^-$	315.07216	315.07226	-0.32	**109.02953**(100), 153.01942(73), 315.07376(4)
**7**	**Hydroxybenzoic acid**	1.37	$C_7H_5O_3^-$	137.02442	137.02444	-0.17	**93.03456**(100), 137.02449(22)
**8**	**Di-O-hydroxybenzoyl hexoside**	5.81	$C_{20}H_{19}O_{10}^-$	419.09837	419.09837	0.01	93.03466(15), **137.02451**(100), 281.06714(4)
**9**	**Dihydroxybenzoyl-feruloyl hexoside**	5.87	$C_{23}H_{23}O_{12}^-$	491.11950	491.11965	-0.31	**152.01160**(100), 153.01938(43), 161.02446(29), 175.04005(49), 193.05109(11), 315.07263(45)
**10**	**Hydroxybenzoyl-galloyl hexoside**	6.07	$C_{20}H_{19}O_{12}^-$	451.08820	451.08812	0.18	93.03426(4), 125.02456(16), 137.02455(87), 168.00655(82), 169.01410(12), **331.06741**(100)
Hydroxycinnamic acid derivatives							
**11**	**3-O-Caffeoylquinic acid**	0.75	$C_{16}H_{17}O_9^-$	353.08781	353.08739	1.18	135.04517(26), 161.02467(4), 173.04526(3), 179.03496(67), **191.05608**(100)
**12**	**5-O-Caffeoylquinic acid**	1.19	$C_{16}H_{17}O_9^-$	353.08781	353.08750	0.86	**191.05626**(100)
**13**	**Coumaroyl hexoside**	1.45	$C_{15}H_{17}O_8^-$	325.09289	325.09291	-0.06	119.05035(59), 145.02974(39), **163.04024**(100), 193.05063(12)
**14**	**Caffeic acid**	1.71	$C_9H_7O_4^-$	179.03498	179.03493	0.30	**135.04532**(100), 179.03528(19)
**15**	**Aesculetin**	1.75	$C_9H_5O_4^-$	177.01933	177.01933	0.04	105.03462(5), 133.02959(12), **177.01930**(100)
**16**	**3-O-Caffeoylshikimic acid**	3.86	$C_{16}H_{15}O_8^-$	335.07724	335.07723	0.03	135.04518(61), 161.02452(22), **179.03510**(100)

**17**	**Coumaric acid**	4.11	$C_9H_7O_3^-$	163.03950	163.03980	-1.84	**119.05034**(100)
**18**	**Caffeoyl hexoside**	4.12	$C_{15}H_{17}O_9^-$	341.08781	341.08768	0.38	109.02956(26), **135.04524**(100)
**19**	**4-O-Feruloylquinic acid**	4.28	$C_{17}H_{19}O_9^-$	367.10346	367.10328	0.48	111.0453(10), 134.03757(10), **173.04564**(100), 191.05626(98), 193.05077(28),
**20**	**Dan Shen Suan C**	4.23	$C_{18}H_{17}O_9^-$	377.08781	377.08759	0.56	135.04532(35), 153.09227(54), **161.02457**(100), 179.03511(21), 197.04558(43)
**21**	**5-O-Coumaroylquinic acid**	4.57	$C_{16}H_{17}O_8^-$	337.09289	337.09319	-0.90	111.0089(28), 161.09732(6), 163.07658(11), 173.04578(3), 179.10722(5), **191.05617**(100)
**22**	**Yunnaneic acid E**	4.66	$C_{27}H_{23}O_{14}^-$	571.10933	571.10945	-0.21	109.02947(22), **135.04521**(100), 179.03506(32), 197.04565(53), 215.10797(14), 241.08705(27)
**23**	**Sinapic acid**	4.72	$C_{11}H_{11}O_5^-$	223.06120	223.06119	0.02	149.02431(32), 163.03969(15), 164.04784(93), 193.01418(37), **208.03764**(100), 223.06050(14)
**24**	**Salvianolic acid K**	4.92	$C_{27}H_{23}O_{13}^-$	555.11442	555.11440	0.02	101.02440(15), 135.04518(24), 161.02490(21), 179.03516(59), 197.04544(18), **295.06146**(100)
**25**	**Dicaffeoyltartaric (chicoric) acid**	4.95	$C_{22}H_{17}O_{12}^-$	473.07255	473.07240	0.31	135.04526(12), 149.00926(95), 161.02487(3), **179.03506**(100), 219.03004(10)
**26**	**Caffeoyltartaric (caftaric) acid**	4.97	$C_{13}H_{11}O_9^-$	311.04086	311.04102	-0.51	135.04524(40), **149.00925**(100), 179.03519(89)
**27**	**Yunnaneic acid F**	5.15	$C_{29}H_{25}O_{14}^-$	597.12498	597.12475	0.39	**135.04527**(100), 179.03514(42), 197.04568(76), 267.10269(79), 293.08209(27), 311.09293(28)
**28**	**Salviaflaside**	5.15	$C_{24}H_{25}O_{13}^-$	521.13007	521.13030	-0.45	135.0453(41), **161.02454**(100), 179.0352(39), 197.0457(39), 323.07748(7), 329.13956(13)
**29**	**Yunnaneic acid D**	5.16	$C_{27}H_{23}O_{12}^-$	539.11950	539.12013	-1.18	**101.02446**(100), 113.02453(46), 135.04538(40), 161.04597(64), 253.08704(18), 331.17651(62)
**30**	**Ferulic acid**	5.18	$C_{10}H_9O_4^-$	193.05063	193.05066	-0.14	**134.03737**(100), 149.06107(7), 178.02716(27), 193.05064(8)
**31**	**3,4-Dicaffeoylquinic acid**	5.47	$C_{25}H_{23}O_{12}^-$	515.11950	515.11949	0.03	135.04526(14), 173.04567(16), 179.0351(73), **191.05626**(100), 353.08798(7)
**32**	**Sagerinic acid**	5.60	$C_{36}H_{31}O_{16}^-$	719.16176	719.16173	0.04	**161.02448**(100), 179.0351(10), 197.04564(40)
**33**	**Lithospermic acid hexoside**	5.63	$C_{33}H_{31}O_{17}^-$	699.15667	699.15601	0.95	135.04529(34), 149.02452(16), **161.02454**(100), 177.01950(10), 179.03514(23), 197.04564(49)
**34**	**4,5-Dicaffeoylquinic acid**	5.68	$C_{25}H_{23}O_{12}^-$	515.11950	515.11931	0.37	135.04518(12), **173.04564**(100), 179.03505(81), 191.05626(31), 353.08762(12)

**35**	**Rosmarinic acid**	5.72	$C_{18}H_{15}O_8^-$	359.07724	359.07672	1.45	135.04521(7), **161.02448**(100), 179.03508(20), 197.04568(30)
**36**	**Cleroden D**	5.75	$C_{36}H_{31}O_{15}^-$	703.16684	703.16655	0.41	135.04523(8), **161.02452**(100), 179.03500(10), 181.05072(11), 197.04568(22)
**37**	**Orthosiphoic acid A**	5.98	$C_{27}H_{21}O_{11}^-$	521.10894	521.10912	-0.35	101.02444(17), 119.05029(45), 145.02954(19), **163.04015**(100), 281.04565(17), 359.07697(33)
**38**	**Lithospermic acid**	6.01	$C_{27}H_{21}O_{12}^-$	537.10385	537.10398	-0.24	135.04524(45), **161.02449**(100), 177.01944(11), 179.03508(19), 197.04564(42)
**39**	**Salvianolic acid**	6.01	$C_{26}H_{21}O_{10}^-$	493.11402	493.11417	-0.31	133.02972(4), 135.04523(48), **161.02448**(100), 179.03496(18), 197.04561(35)
**40**	**Methyl rosmarinate**	6.20	$C_{19}H_{17}O_8^-$	373.09289	373.09276	0.35	**135.04529**(100), 160.01678(16), 175.04015(72), 179.03517(34), 197.04573(48)
**41**	**Caffeoyl-feruloyl dihexoside**	6.24	$C_{31}H_{35}O_{17}^-$	679.18797	679.18840	-0.63	109.02962(14), 134.03748(25), 149.06097(9), 175.04015(74), **193.05074**(100)
**42**	**Salvianolic acid B**	6.29	$C_{36}H_{29}O_{16}^-$	717.14611	717.14659	-0.68	135.0453(5), 161.02466(4), 179.03505(4), 295.06149(5), 321.04062(11), **339.05075**(100)
**43**	**Sagecoumarin**	6.39	$C_{27}H_{19}O_{12}^-$	535.08820	535.08854	-0.63	135.04526(18), 161.02449(53), **177.01935**(100), 179.03502(20), 197.04561(16), 311.05637(8)
**44**	**Salvianolic acid C**	6.57	$C_{26}H_{19}O_{10}^-$	491.09837	491.09868	-0.63	**135.04529**(100), 179.03505(4), 311.05603(3), 329.23334(4)
**45**	**Yunnaneic acid H**	6.61	$C_{36}H_{25}O_{16}^-$	713.11481	713.11539	-0.81	161.02455(7), 179.03508(7), 311.05649(4), **335.01984**(100), 357.09726(3), 551.17749(6)
**46**	**Schizotenuin A**	6.64	$C_{36}H_{27}O_{16}^-$	715.13046	715.13081	-0.49	135.04523(45), 179.03506(5), 293.04581(4), **311.05615**(100), 337.03534(3)
**47**	**Methyl lithospermate**	6.81	$C_{28}H_{23}O_{12}^-$	551.11950	551.11967	-0.30	135.04526(32), **161.02451**(100), 179.03505(18), 193.05077(39), 197.04573(26)
**48**	**Dimethyl lithospermate**	6.88	$C_{29}H_{25}O_{12}^-$	565.13515	565.13539	-0.43	135.04527(74), 151.04024(16), 161.02457(97), 179.03513(28), 197.04576(21), **207.06641**(100)
**49**	**Nepetoidin A or B**	6.93	$C_{17}H_{13}O_6^-$	313.07176	313.07191	-0.48	133.02965(3), 151.04022(8), **161.02451**(100)
**50**	**Lycopic acid C**	7.16	$C_{27}H_{19}O_{11}^-$	519.09329	519.09352	-0.45	**135.04527**(100), 161.02452(40), 177.01949(6), 179.03545(9), 311.05627(19), 339.05084(43)
Iridoid glycosides							

**51**	**Loganin**	4.64	$C_{17}H_{25}O_{10}^{-}$	389.14532	389.14545	-0.32	**101.02445**(100), 119.03498(8), 125.02436(18), 157.05055(25), 209.08311(9), 227.09207(7)
**52**	**Alpigenoside**	4.65	$C_{18}H_{27}O_{12}^{-}$	435.15080	435.15041	0.91	89.02451(54), **101.02450**(100), 113.02488(9), 119.0353(16), 157.05089(63)
**53**	**Geniposide**	4.84	$C_{17}H_{23}O_{10}^{-}$	387.12967	387.12944	0.58	**101.02445**(100), 123.04519(24), 155.03494(16), 181.0507(24), 193.05051(14)
**54**	**5-Deoxylamiol**	5.07	$C_{16}H_{25}O_{9}^{-}$	361.15041	361.15074	-0.94	135.04539(25), **161.02466**(100), 163.02997(68), 179.03517(21), 181.03839(16), 199.05063(30)
**55**	**6′-O-Coumaroyl-8-epikingiside**	5.39	$C_{26}H_{29}O_{13}^{-}$	549.16137	549.16131	0.10	119.05032(9), **125.02453**(100), 145.02959(94), 163.04005(32), 307.08234(14)
**56**	**2′-O-(3"-Hydroxybenzoyl)-8-epikingiside**	5.40	$C_{24}H_{27}O_{13}^{-}$	523.14572	523.14529	0.82	101.02458(13), 113.02448(16), 119.03503(17), **137.02454**(100), 161.02458(4), 179.03479(4)
**57**	**1-O-Hexosyl-loganic acid (Asystasioside A)**	5.40	$C_{22}H_{33}O_{14}^{-}$	521.18758	521.18753	0.09	**89.02448**(100), 119.03513(40), 161.04584(4), 179.05594(10), 197.04553(9), 359.09839(4)
**58**	**3'-O-Caffeoyl sweroside**	5.56	$C_{25}H_{27}O_{12}^{-}$	519.15080	519.15080	0.00	125.02451(98), 135.04532(18), 145.02956(12), **161.02457**(100), 179.03496(21), 323.07736(22)
**59**	**Deoxygeniposide**	5.84	$C_{17}H_{23}O_{9}^{-}$	371.13476	371.13463	0.34	165.09212(4), **209.08194**(100)
**60**	**Coumaroyl-secologanoside**	5.88	$C_{25}H_{27}O_{13}^{-}$	535.14572	535.14628	-1.06	121.06597(13), **145.02960**(100), 161.02486(8), 163.04016(20), 177.01976(7), 205.05067(14)
**61**	**7-O-Coumaroyl-loganic acid (Linearoside; Gentiana)**	6.01	$C_{25}H_{29}O_{12}^{-}$	521.16645	521.16695	-0.96	119.05032(49), 145.02953(17), 161.0457(8), **163.04015**(100), 281.04578(12), 359.07718(11)
**62**	**Caffeoyl-loganin**	6.19	$C_{26}H_{31}O_{13}^{-}$	551.17702	551.17721	-0.35	101.02441(8), 135.04523(20), 161.02451(7), **179.03502**(100), 329.10315(5), 357.09781(34)
**63**	**Hydroxybenzoyl-loganin**	6.34	$C_{24}H_{29}O_{12}^{-}$	509.16645	509.16631	0.28	101.02444(19), **137.02448**(100), 161.02451(37), 197.04565(18)
**64**	**Coumaroyl-loganin**	6.51	$C_{26}H_{31}O_{12}^{-}$	535.18210	535.18222	-0.23	101.02444(25), 119.05032(20), 135.04527(8), 161.02452(16), **163.04016**(100), 177.01938(31)
**65**	**Feruloyl-loganin**	6.59	$C_{27}H_{33}O_{13}^{-}$	565.19267	565.19303	-0.64	134.03745(20), 149.06087(8), **193.05069**(100)
Flavan-3-ol gallates							
**66**	**Gallocatechin 3-O-gallate**	5.42	$C_{22}H_{17}O_{11}^{-}$	457.07764	457.07746	0.39	125.02460(23), **169.01421**(100), 305.06714(5)
**67**	**Catechin 3-O-gallate**	6.03	$C_{22}H_{17}O_{10}^{-}$	441.08272	441.08259	0.30	125.02464(28), **169.01428**(100), 245.08339(14), 289.07190(26)

**68**	**Epicatechin 3-O-gallate**	6.27	$C_{22}H_{17}O_{10}^{-}$	441.08272	441.08261	0.26	125.02483(23), **169.01410**(100), 245.08315(9), 289.07294(23)
**69**	**Epgalloicatechin 3-O-gallate**	6.64	$C_{22}H_{17}O_{11}^{-}$	457.07764	457.07764	-0.02	125.02454(29), **169.01433**(100)
Flavonoid O-glycosides							
**70**	**Myricetin 3-O-hexoside**	4.56	$C_{21}H_{19}O_{13}^{-}$	479.08311	479.08297	0.30	178.99857(2), **316.02252**(100), 317.02994(13)
**71**	**Quercetin 3-O-(6"-rhamnosyl)-hexoside-7-O-rhamnoside**	4.65	$C_{33}H_{39}O_{20}^{-}$	755.20402	755.20395	0.08	**300.02759**(100), 301.03464(8)
**72**	**Kaempferol 3-O-(6"-rhamnosyl)-hexoside-7-O-rhamnoside**	4.95	$C_{33}H_{39}O_{19}^{-}$	739.20910	739.20926	-0.22	284.03281(65), 285.04068(46), 327.05203(4), 430.09064(30), 431.09875(5), **593.15167**(100)
**73**	**Quercetin 3-O-(6"-rhamnosyl)-hexoside (Rutin)**	4.97	$C_{27}H_{29}O_{16}^{-}$	609.14611	609.14620	-0.15	**300.02768**(100), 301.03531(38)
**74**	**Kaempferol 3-O-hexoside-7-O-hexuronide**	5.08	$C_{27}H_{27}O_{17}^{-}$	623.12537	623.12649	-1.79	119.03518(24), 161.02463(44), 179.05594(20), 221.06693(11), **285.04077**(100), 447.09384(82)
**75**	**Quercetin 3-O-hexuronide**	5.11	$C_{21}H_{17}O_{13}^{-}$	477.06746	477.06783	-0.77	137.02449(51), 151.00368(10), 153.01945(8), 161.02454(11), 178.99861(9), **301.03543**(100)
**76**	**Quercetin 3-O-hexoside**	5.13	$C_{21}H_{19}O_{12}^{-}$	463.08820	463.08825	-0.12	151.00395(3), 178.99829(3), 271.02524(3), **300.02759**(100), 301.03531(37)
**77**	**Kaempferol 3-O-hexoside**	5.21	$C_{21}H_{19}O_{11}^{-}$	447.09329	447.09334	-0.13	284.0329(34), **285.04071**(100)
**78**	**Kaempferol 3-O-(6"-rhamnosyl)-hexoside**	5.22	$C_{27}H_{29}O_{15}^{-}$	593.15119	593.15119	0.01	255.03046(3), 284.03284(79), **285.04062**(100)
**79**	**Naringenin 7-O-(2"-rhamnosyl)-hexoside (Naringin)**	5.52	$C_{27}H_{31}O_{14}^{-}$	579.17193	579.17142	0.88	119.05044(5), 151.00369(59), **271.06116**(100), 313.07065(5), 339.0715(4), 459.11563(17)
**80**	**Isorhamnetin 3-O-(6"-rhamnosyl)-hexoside**	5.34	$C_{28}H_{31}O_{16}^{-}$	623.16176	623.16116	0.96	299.01990(8), 300.02734(10), 314.04315(50), **315.05090**(100)
**81**	**Quercetin 3-O-pentoside**	5.36	$C_{20}H_{17}O_{11}^{-}$	433.07764	433.07702	1.43	271.02533(3), **300.02777**(100), 301.03558(19)
**82**	**Quercetin 3-O-(2"-caffeoyl-6"-rhamnosyl)-hexoside-7-O-rhamnoside**	5.37	$C_{42}H_{45}O_{23}^{-}$	917.23571	917.23671	-1.08	135.04529(3), **300.02771**(100), 301.03534(25), 446.08575(11), 609.1463(27), 755.20288(29)
**83**	**Quercetin 3-O-rhamoside**	5.45	$C_{21}H_{19}O_{11}^{-}$	447.09329	447.09313	0.35	151.00378(6), 178.99875(4), 271.0246(3), **300.02756**(100), 301.03528(66)
**84**	**Isorhamnetin 3-O-hexoside**	5.55	$C_{22}H_{21}O_{12}^{-}$	477.10385	477.10340	0.95	243.02965(5), 271.02487(8), 285.04074(8), 286.04837(7), **314.04318**(100), 315.05063(11)

**85**	**Quercetin 3-O-(2"-coumaroyl-6"-rhamnosyl)-hexoside-7-O-rhamnoside**	5.57	$C_{42}H_{45}O_{22}^{-}$	901.24080	901.24106	-0.29	**300.02774**(100), 301.03525(31), 446.08569(21), 447.09198(4), 609.14624(31), 755.19025(24)
**86**	**Apigenin 7-O-hexoside**	5.58	$C_{21}H_{19}O_{10}^{-}$	431.09837	431.09829	0.20	**268.03775**(100), 269.04544(59), 311.05615(13)
**87**	**Chrysoeriol 7-O-(6"-rhamnosyl)-hexoside**	5.63	$C_{28}H_{31}O_{15}^{-}$	607.16684	607.16696	-0.19	284.03311(24), **299.05627**(100)
**88**	**Hesperetin 7-O-(6"-rhamnosyl)-hexoside (Hesperidin)**	5.77	$C_{28}H_{33}O_{15}^{-}$	609.18249	609.18254	-0.08	273.07700(8), 286.05057(6), **301.07217**(100)
**89**	**Luteolin 7-O-hexuronide**	5.78	$C_{21}H_{17}O_{12}^{-}$	461.07255	461.07250	0.11	**285.04053**(100)
**90**	**Kaempferol 3-O-(2"-rhamnosyl-6"-coumaroyl)-hexoside-7-O-rhamnoside**	6.04	$C_{42}H_{45}O_{21}^{-}$	885.24588	885.24624	-0.41	**284.03281**(100), 285.04053(65), 363.18073(4), 575.14087(4), 593.15149(41), 739.18835(64)
**91**	**Chrysoeriol 7-O-hexoside**	6.54	$C_{22}H_{21}O_{11}^{-}$	461.10894	461.10900	-0.13	284.03250(20), **299.05621**(100)
Flavonoid C-glycosides							
**92**	**Apigenin 6-C-pentoside-8-C-hexoside**	4.68	$C_{26}H_{27}O_{14}^{-}$	563.14063	563.14016	0.84	**353.06647**(100), 383.07718(83), 413.08752(15), 443.0986(45), 473.10925(36), 563.14001(37)
**93**	**Apigenin 6-C-rhamnoside-8-C-hexoside**	5.05	$C_{27}H_{29}O_{14}^{-}$	577.15628	577.15637	-0.16	**293.04559**(100), 323.05682(16), 353.06638(48), 383.07770(35), 413.08795(33), 457.11359(32)
**94**	**Apigenin 8-C-hexoside**	5.86	$C_{21}H_{19}O_{10}^{-}$	431.09837	431.09828	0.21	283.06100(17), **311.05612**(100), 323.05606(3), 341.06717(10)
Flavonoid aglycones							
**95**	**Kaempferol**	6.27	$C_{15}H_{9}O_{6}^{-}$	285.04046	285.04050	-0.14	151.00374(2), 175.04007(1), **285.04047**(100)
**96**	**Quercetin**	6.31	$C_{15}H_{9}O_{7}^{-}$	301.03538	301.03543	-0.18	107.01389(7), 121.02953(16), **151.00369**(100), 178.9986(49), 273.04044(10), 301.03543(67)
**97**	**Myricetin**	6.53	$C_{15}H_{9}O_{8}^{-}$	317.03029	317.03011	0.56	137.02422(12), **151.00363**(100), 165.05566(36), 177.01868(7), 178.99863(14), 317.03009(15)
**98**	**Naringenin**	6.69	$C_{15}H_{11}O_{5}^{-}$	271.06120	271.06135	-0.56	93.03483(11), 107.01386(14), 119.05029(41), **151.00369**(100), 177.01973(12), 271.06125(45)
**99**	**Apigenin**	6.71	$C_{15}H_{9}O_{5}^{-}$	269.04555	269.04564	-0.36	151.00389(4), 225.05585(2), **269.04550**(100)
**100**	**Thymusin**	6.75	$C_{17}H_{13}O_{7}^{-}$	329.06668	329.06653	0.45	299.01987(72), **314.04346**(100), 329.06696(31)
**101**	**Chrysoeriol**	6.75	$C_{16}H_{11}O_{6}^{-}$	299.05611	299.05617	-0.20	**284.03275**(100), 299.05664(7)
**102**	**Isorhamnetin**	6.88	$C_{16}H_{11}O_{7}^{-}$	315.05103	315.05126	-0.76	151.00337(2), **300.02765**(100), 315.05118(55)
**103**	**Cirsimaritin**	7.39	$C_{17}H_{13}O_{6}^{-}$	313.07176	313.07199	-0.72	269.04688(9), **283.02499**(100), 298.04807(89)

**104**	**Rhamnetin**	7.39	$C_{16}H_{11}O_7^-$	315.05103	315.05128	-0.81	**165.01945**(100), 193.01410(14), 287.05643(9), 300.02798(23), 315.05133(34)
**105**	**Acacetin**	7.89	$C_{16}H_{11}O_5^-$	283.06120	283.06137	-0.60	268.03781(93), **283.06122**(100)
**106**	**Hispidulin**	7.93	$C_{16}H_{11}O_6^-$	299.05611	299.05617	-0.20	165.01939(49), 243.06633(11), 256.03793(8), **271.06131**(100), 284.03278(36), 299.05624(38)
Xanthone glycosides							
**107**	**Dihydroxy-tetramethoxy xanthone O-pentosyl-hexoside**	6.25	$C_{28}H_{33}O_{17}^-$	641.17232	641.17299	-1.03	302.00632(3), 317.03033(67), **332.05389**(100), 347.07672(16)
**108**	**Dihydroxy-dimethoxy xanthone O-hexoside**	6.35	$C_{21}H_{21}O_{11}^-$	449.10894	449.10915	-0.47	257.00830(4), **272.03268**(100), 287.05627(79)
**109**	**Trihydroxy-methoxy xanthone O-hexoside (Swertianolin)**	6.35	$C_{20}H_{19}O_{11}^-$	435.09329	435.09329	0.00	**137.02448**(100), 152.01157(75), 258.01868(2), 273.04053(62), 297.06177(17), 315.07217(35)
**110**	**Trihydroxy-dimethoxy xanthone O-pentosyl-hexoside**	6.36	$C_{26}H_{29}O_{16}^-$	597.14611	597.14540	1.19	273.00415(6), 288.02780(36), **303.05124**(100)
**111**	**Trihydroxy-methoxy xanthone O-pentosyl-hexoside**	6.36	$C_{25}H_{27}O_{15}^-$	567.13554	567.13573	-0.33	258.01694(9), **273.04065**(100)
**112**	**Trihydroxy-dimethoxy xanthone O-rhamnosyl-hexoside**	6.42	$C_{27}H_{31}O_{16}^-$	611.16176	611.16218	-0.69	273.00415(6), 288.0278(32), **303.05121**(100)
**113**	**Tetrahydroxy-dimethoxy xanthone O-hexoside**	6.53	$C_{23}H_{25}O_{13}^-$	509.13007	509.12952	1.07	**161.02451**(100), 179.03508(15), 302.00696(14), 317.03043(98), 332.05402(84), 347.07730(8)
**114**	**Trihydroxy-dimethoxy xanthone O-hexoside**	6.60	$C_{21}H_{21}O_{12}^-$	465.10385	465.10385	0.00	273.00446(19), 288.02786(70), **303.05130**(100)
**115**	**Trihydroxy-dimethoxy xanthone O-caffeoyl-hexoside**	6.86	$C_{30}H_{27}O_{15}^-$	627.13554	627.13617	-1.00	135.04529(8), 161.02469(15), 179.03525(6), 273.00385(10), 288.02777(43), **303.05127**(100)
Xanthone aglycones							
**116**	**Trihydroxy-dimethoxy xanthone**	6.43	$C_{15}H_{11}O_7^-$	303.05103	303.05103	-0.02	259.02618(4), **273.00421**(100), 288.02768(80), 303.05087(26)
**117**	**Trihydroxy-methoxy xanthone**	7.05	$C_{14}H_9O_6^-$	273.04046	273.04066	-0.73	258.01721(95), **273.04083**(100)
**118**	**Dihydroxy-dimethoxy xanthone (Gentiacaulin)**	7.89	$C_{15}H_{11}O_6^-$	287.05611	287.05632	-0.74	257.00931(11), **272.03268**(100), 287.056240(7)
**119**	**Trihydroxy-methoxy xanthone (Swertianin)**	7.99	$C_{14}H_9O_6^-$	273.04046	273.04059	-0.47	**258.01700**(100), 273.04047(29)

**120**	**Trihydroxy-dimethoxy xanthone**	8.03	$C_{15}H_{11}O_7^-$	303.05103	303.05130	-0.91	**273.00433**(100), 288.02783(77), 303.05133(22)
**121**	**Trihydroxy-dimethoxy xanthone**	8.56	$C_{15}H_{11}O_7^-$	303.05103	303.05134	-1.04	273.00430(32), **288.02780**(100), 303.05460(11)
