# Supplementary material for: A Chemically Characterized Polyherbal Extract Selectively Attenuates Chronic Unpredictable Stress-Induced Redox-Sensitive, Inflammatory, and Metabolic Alterations in Female Rats
Source: Int J Mol Sci. 2026 Sep 17;27(18):8298. doi: 10.3390/ijms27188298 (PMC13607128; doi:10.3390/ijms27188298)
Supplement: Supplementary file 1 [file ijms-27-08298-s001.zip › Supplementary Note S1.pdf]

Supplementary Note S1. Comprehensive phytochemical characterization of the polyherbal extract by UHPLC-Orbitrap HRMS

UHPLC-Orbitrap HRMS analysis enabled the tentative identification of **121 metabolites** belonging to nine major phytochemical classes. Compound annotation was based on retention time, accurate mass measurements, elemental composition, mass accuracy, and characteristic MS/MS fragmentation patterns. The low mass deviations, generally within ± 2 ppm, support high confidence in the proposed elemental compositions. A complete list of identified metabolites together with their analytical characteristics is provided in **Supplementary Table S1**.

Hydroxybenzoic acid derivatives

Hydroxybenzoic acid derivatives contributed to the phenolic profile. Their fragmentation patterns were characterized by ions corresponding to hydroxybenzoic, dihydroxybenzoic, gallic, or vanillic acid-related moieties.

Hydroxycinnamic acid derivatives

Hydroxycinnamic acid derivatives represented the largest individual group, comprising 40 compounds. This class included relatively simple molecules, such as caffeic, coumaric, ferulic, and sinapic acids, together with caffeoylquinic acids, caffeoylshikimic acid, caffeoyltartaric acid, dicaffeoyltartaric acid, dicaffeoylquinic acid isomers, rosmarinic acid, lithospermic acid, salvianolic acids, yunnaneic acids, and several methylated or glycosylated derivatives. The abundance and structural diversity of these compounds indicate that the extract contains an extensive network of caffeic acid-derived metabolites rather than being dominated by a single phenolic marker.

Iridoid glycosides

A second prominent chemical signature was provided by iridoid glycosides. Fifteen compounds were assigned to this group, including loganin, geniposide, alpigenoside, 5-deoxylamiol, deoxygeniposide, loganic acid derivatives, and several acylated loganin, sweroside, secologanoside, epikingiside, and loganic acid conjugates. The occurrence of caffeoyl-, coumaroyl-, feruloyl-, and hydroxybenzoyl-substituted iridoids indicates considerable structural diversity within this class.

Xanthone derivatives

Nine xanthone glycosides and six xanthone aglycones were tentatively identified. These included swertianolin, swertianin, gentiacaulin, and a range of hydroxylated and methoxylated xanthone derivatives carrying hexose, pentose, rhamnose, or caffeoyl-hexose residues. The simultaneous detection of both glycosylated and non-glycosylated forms indicates that the extract contains xanthones over a broad polarity range. Glycosylated xanthones would be expected to be efficiently recovered by 70% ethanol, whereas the same solvent system can also extract moderately lipophilic methoxylated aglycones.

Flavonoids

Flavonoids represented another major portion of the extract. Twenty-two flavonoid O-glycosides, three flavonoid C-glycosides, and twelve flavonoid aglycones were identified. The O-glycosides

were derived mainly from quercetin, kaempferol, myricetin, apigenin, luteolin, isorhamnetin, chrysoeriol, naringenin, and hesperetin. Several mono-, di-, and triglycosylated structures were detected, including rutin, quercetin hexoside, quercetin hexuronide, kaempferol hexoside, isorhamnetin glycosides, apigenin hexoside, luteolin hexuronide, naringin, and hesperidin. The fragmentation of these compounds generally involved neutral losses consistent with hexose, deoxyhexose, pentose, or hexuronic acid residues, producing characteristic aglycone ions. For example, quercetin glycosides generated ions at approximately m/z 300/301, kaempferol derivatives at m/z 284/285, isorhamnetin derivatives at m/z 314/315, and chrysoeriol derivatives at m/z 299. These product ions support the assignment of the corresponding aglycone skeletons. More complex acylated flavonoid glycosides were also detected, including caffeoyl- and coumaroyl-substituted quercetin and kaempferol glycosides. Such structures expand the chemical diversity of the formulation and demonstrate that the extract contains compounds combining flavonoid, sugar, and hydroxycinnamic acid moieties. The identified flavonoid C-glycosides were apigenin-based compounds, including apigenin 8-C-hexoside and mixed pentosyl/hexosyl or rhamnosyl/hexosyl derivatives. Their MS/MS spectra showed cross-ring fragmentation and product ions consistent with C-glycosidic linkages rather than the simple neutral sugar losses typical of O-glycosides. The flavonoid aglycone fraction included quercetin, kaempferol, myricetin, naringenin, apigenin, chrysoeriol, isorhamnetin, acacetin, hispidulin, cirsimaritin, rhamnetin, and thymusin. The presence of both hydroxylated and methoxylated flavonoids indicates a broad range of polarity and structural substitution. Four flavan-3-ol gallates were additionally identified: gallocatechin 3-O-gallate, catechin 3-O-gallate, epicatechin 3-O-gallate, and epigallocatechin 3-O-gallate. Their spectra contained prominent ions attributable to gallic acid or gallate residues.
